# Engineering ‘designer’ glycomodules for boosting recombinant protein secretion in tobacco hairy root culture and studying hydroxyproline‐*O*‐glycosylation process in plants

**DOI:** 10.1111/pbi.13043

**Published:** 2018-12-14

**Authors:** Ningning Zhang, Tristen Wright, Xiaoting Wang, Uddhab Karki, Brett J. Savary, Jianfeng Xu

**Affiliations:** ^1^ Arkansas Biosciences Institute Arkansas State University Jonesboro AR USA; ^2^ College of Agriculture and Technology Arkansas State University Jonesboro AR USA

**Keywords:** hairy roots, recombinant proteins, hydroxyproline‐*O*‐glycosylation, extensins, arabinogalactan proteins, secretion

## Abstract

The key technical bottleneck for exploiting plant hairy root cultures as a robust bioproduction platform for therapeutic proteins has been low protein productivity, particularly low secreted protein yields. To address this, we engineered novel hydroxyproline (Hyp)‐*O*‐glycosylated peptides (HypGPs) into tobacco hairy roots to boost the extracellular secretion of fused proteins and to elucidate Hyp‐*O*‐glycosylation process of plant cell wall Hyp‐rich glycoproteins. HypGPs representing two major types of cell wall glycoproteins were examined: an extensin module consisting of 18 tandem repeats of ‘Ser‐Hyp‐Hyp‐Hyp‐Hyp’ motif or (SP4)_18_ and an arabinogalactan protein module consisting of 32 tandem repeats of ‘Ser‐Hyp’ motif or (SP)_32_. Each module was expressed in tobacco hairy roots as a fusion to the enhanced green fluorescence protein (EGFP). Hairy root cultures engineered with a HypGP module secreted up to 56‐fold greater levels of EGFP, compared with an EGFP control lacking any HypGP module, supporting the function of HypGP modules as a molecular carrier in promoting efficient transport of fused proteins into the culture media. The engineered (SP4)_18_ and (SP)_32_ modules underwent Hyp‐*O*‐glycosylation with arabino‐oligosaccharides and arabinogalactan polysaccharides, respectively, which were essential in facilitating secretion of the fused EGFP protein. Distinct non‐Hyp‐*O*‐glycosylated (SP4)_18_‐EGFP and (SP)_32_‐EGFP intermediates were consistently accumulated within the root tissues, indicating a rate‐limiting trafficking and/or glycosylation of the engineered HypGP modules. An updated model depicting the intracellular trafficking, Hyp‐*O*‐glycosylation and extracellular secretion of extensin‐styled (SP4)_18_ module and AGP‐styled (SP)_32_ module is proposed.

## Introduction

Molecular farming in plants is recognized as an advantageous platform for producing therapeutic proteins through the significant advantages in cost and safety over other eukaryotic systems. Increasing concerns over regulatory compliance and product safety have prompted a resurgence of interest in molecular farming with contained *in vitro* systems such as plant cells and tissues cultured in bioreactors (Santos *et al*., 2016; Xu *et al*., 2011). Hairy roots generated from plant tissue inoculated with *Agrobacterium rhizogenes* strains harbouring a root‐inducing (Ri) plasmid are an attractive culture system as it integrates the merits of plant cell culture with those of whole‐plant cultivation (Georgiev *et al*., 2012). Axenic hairy roots propagate at high rates in controlled environments, free from pathogen contaminants. As a fully differentiated organ, hairy roots provide additional benefits that include genotype and phenotype stability and autotrophy for plant hormones (Georgiev *et al*., 2012; Guillon *et al*., 2006). Despite these benefits, a bottleneck exits in exploiting this *in vitro* technology for commercial purposes due to low protein productivity, particularly low secreted protein yields. Addressing this, the addition of certain chemicals to the culture medium (i.e. KNO_3_, α‐naphthaleneacetic acid and polyvinylpyrrolidone), which induced lateral root formation and morphological changes, resulted in 30‐fold higher secreted yields for the monoclonal antibody M12 expressed in tobacco hairy roots (Hakkinen *et al*., 2014). The addition of polyvinylpyrrolidone also stabilized secreted human erythropoietin in tobacco hairy root culture, leading to a 5.6‐fold increase in protein yield (Gurusamy *et al*., 2017). The opportunity to recover therapeutic proteins from the culture medium in high yields offers a simplified and low‐cost approach for purifying expressed proteins from a well‐defined and protein‐deficient media (Georgiev *et al*., 2012). We seek to exploit an alternative method, engineering novel hydroxyproline (Hyp)‐*O*‐glycosylated peptide (HypGP) modules as molecular carriers, for generally boosting secreted protein yields in hairy root culture.

Hydroxyproline‐*O*‐glycosylated peptide modules are derived from Hyp‐rich glycoproteins (HRGPs) uniquely present in the cell wall of higher plants and green algae (Kieliszewski and Shpak, 2001). There are three major types of HRGPs, including extensins, proline‐rich proteins and arabinogalactan proteins (AGPs) comprising a superfamily of plant cell wall structural glycoproteins that contribute to the extracellular matrix (Tan *et al*., 2012). HRGPs are synthesized *in planta* through intensive post‐translational modifications termed Hyp‐*O*‐glycosylation that involves proline (Pro) hydroxylation and subsequent *O*‐glycosylation on the Hyp residues. Our earlier work with synthetic genes encoding various signature HRGP‐glycomodule sequences was formative in elucidating the Hyp‐*O*‐glycosylation ‘code’ that defines the determination of peptide sequence on the glycosylation pattern (Kieliszewski and Shpak, 2001; Shpak *et al*., 1999, 2001; Tan *et al*., 2003; Xu *et al*., 2008). These studies revealed Pro residues in repetitive Pro‐rich motifs like ‘Ser‐Pro‐Pro‐Pro‐Pro’ or ‘X‐Pro‐X‐Pro’ (where X is Ser, Thr or Ala) are often hydroxylated. Furthermore, contiguous Hyp residues, such as in extensin‐like ‘Ser‐Hyp‐Hyp‐Hyp‐Hyp’ motifs, are preferred sites of oligo‐arabinosylation. Non‐contiguous Hyp residues, especially when clustered as in AGP‐like ‘X‐Hyp‐X‐Hyp’ motifs, are preferred sites of branched arabinogalactan polysaccharide addition.

Based on the elucidation of the Hyp‐*O*‐glycosylation ‘code’, the utility to design and engineer HypGPs in plant suspension cell cultures has been demonstrated (Kieliszewski *et al*., 2015a,b). Interestingly, engineered designer HypGP modules such as the tandem repeats of ‘Ser‐Pro’ motif or (SP)_*n*_ (*n* = 5, 10, 20, 32) could dramatically increase secreted yields of fused proteins. This was demonstrated for the enhanced green fluorescence protein (EGFP) and two other therapeutic proteins (human growth hormone and interferon) with up to 500× fold increased culture yields (Xu *et al*., 2007, 2010). The function of the engineered HypGPs as a molecular carrier was hypothesized to promote efficient transport of the fused proteins across the plasmalemma and for protecting the proteins from proteolytic degradation (Zhang *et al*., 2016b). Besides higher plant cells, engineering HypGPs comprised of a (SP)_*n*_ motif (*n* = 10, 20) in green microalgae (*Chlamydomonas reinhardtii*) was recently reported to increase the secreted yields of a reporter protein by up to 12‐fold (Ramos‐Martinez *et al*., 2017). However, the precise process for Hyp‐*O*‐glycosylation of the designer HypGPs, particularly the AGPs‐like modules that undergo Hyp‐*O*‐glycosylation with complex polysaccharides in plant cells, is poorly understood (Showalter and Basu, 2016b). The broader applicability of this technology in fully differentiated plants or plant organs has not been explored.

The purposes of this study were: (i) to engineer HypGPs in a differentiated plant organ – hairy roots – for boosting secreted protein yields; and (ii) to use hairy root culture – *in vitro* cultured plant organs – as a novel platform to study the Hyp‐*O*‐glycosylation process of HRGPs *in planta*. HypGPs representing two major types of HRGPs, an extensin module consisting of 18 tandem repeats of ‘Ser‐Hyp‐Hyp‐Hyp‐Hyp’ motif or (SP4)_18_ and an AGP module consisting of 32 tandem repeats of ‘Ser‐Hyp’ motif or (SP)_32_, were each engineered into tobacco hairy roots as a fusion to the reporter protein (EGFP) to facilitate protein detection and purification. The Hyp‐*O*‐glycosylation of engineered HypGP modules and their impacts on the accumulation of fused EGFP in culture media and root tissues were described.

## Results

### Generation of transgenic tobacco hairy root cultures

**Figure 1:**
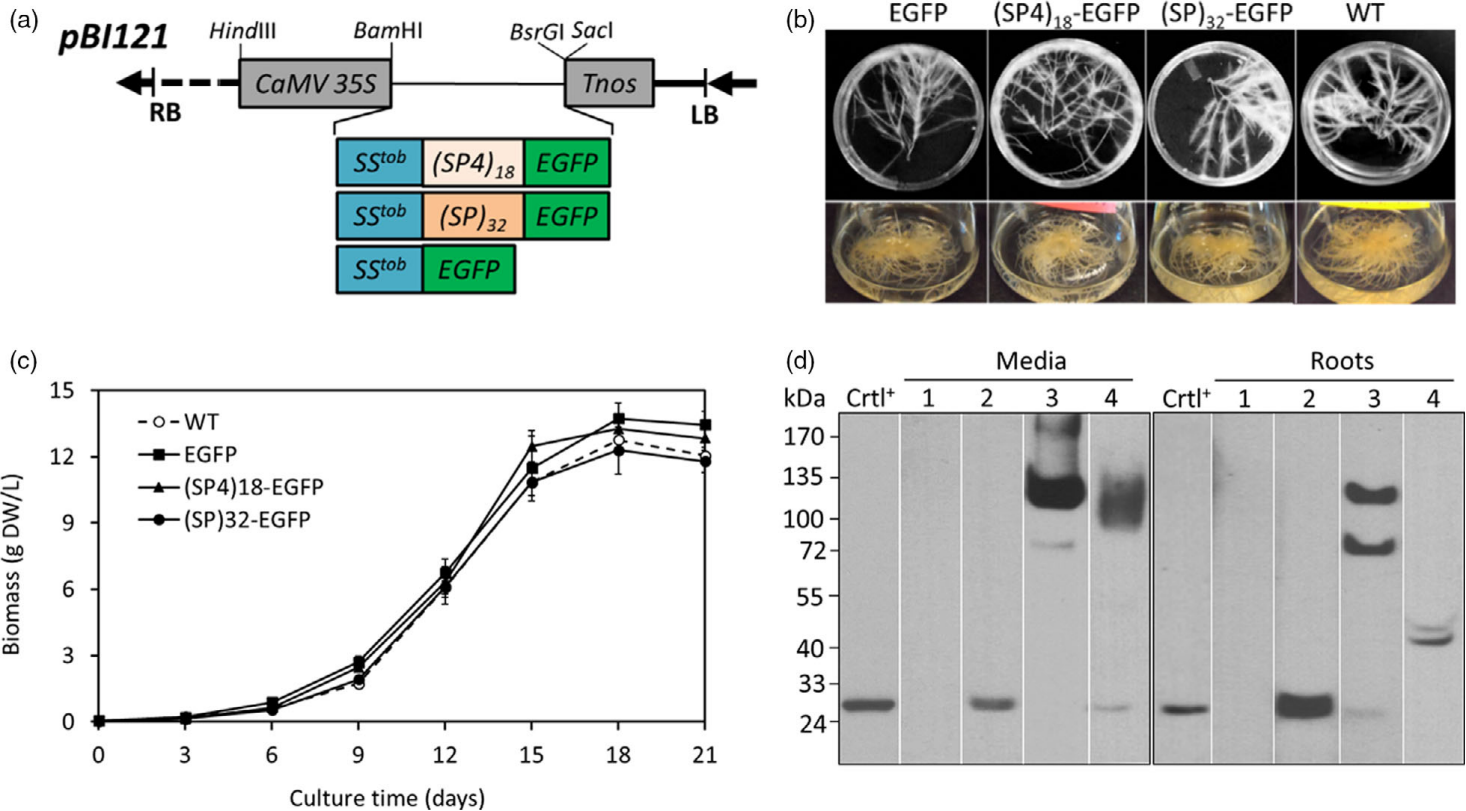
Characterization of transgenic hairy roots expressing EGFP, (SP)_32_‐EGFP and (SP4)_18_‐EGFP. (a) Schematic representation of the *pBI121* binary vectors used to express (SP)_32_‐EGFP, (SP4)_18_‐EGFP and EGFP control in tobacco plants and hairy roots; (b) Hairy roots grown on plates (top) and in liquid media (bottom) for 12 days; (c) Time course of hairy root growth in liquid SH media. Dry weight (DW) of the cultured roots was used to estimate root biomass. The error bars represent the standard deviation of three parallel cultures (three independent flasks); (d) Detection of *EGFP*,* (SP4)*_*18*_ *‐EGFP and (SP)*_*32*_ *‐EGFP* transgene products in hairy roots culture by anti‐EGFP Western blotting. The roots and media were harvested after 12 days of culture. Fifteen μL of culture media or 10 μL clarified root extracts (supernatants) was loaded into each well. The EGFP medium was concentrated by 10‐fold before loading. Crtl^+^: EGFP standard (50 ng); Lane 1, 2, 3, 4 represents the wild‐type hairy root and hairy root expressing EGFP, (SP4)_18_‐EGFP and (SP)_32_‐EGFP respectively.

Each of the three gene constructs encoding (SP4)_18_‐EGFP, (SP)_32_‐EGFP and EGFP control (Figure 1a) was stablely transformed into tobacco plants to first generate transgenic plantlets. The expected molecular size of the three constructs when expressed *in planta* is: ~66, ~113 and 27 kDa respectively (Table S1). Anti‐EGFP Western blotting detected the expression of the three gene constructs in the plantlet leaves (Figure S1). The accumulation levels of EGFP and EGFP *equivalent* of (SP4)_18_‐EGFP and (SP)_32_‐EGFP were 27.5 ± 0.8 μg/g FW, 37.2 ± 2.3 μg/g FW and 32.6 ± 0.9 μg/g FW respectively (*n* = 3 top‐expression transformants; FW = fresh weight). There was a small but significant (*P *<* *0.05) increase in the EGFP accumulation in the (SP4)_18_‐EGFP and (SP)_32_‐EGFP plantlets compared with the plantlets expressing EGFP control.

Transgenic hairy roots were then induced from leaves of the high‐expression plantlets expressing (SP4)_18_‐EGFP, (SP)_32_‐EGFP or EGFP control. At least five high‐expression root lines for each gene construct were selected by anti‐EGFP Western blotting assay (data not shown). The obtained hairy roots were confirmed for T‐DNA integration by PCR detection of the *rolC*,* aux1* and *virD2* genes, which showed positive bands for *rolC* and *aux1*, and a negative result for *virD2* (Figure S2). The transgenic hairy roots were not phenotypically different from the wild‐type ones, all exhibiting typical morphology of hairy roots, including extensive lateral branching and fast growing in plant growth regulator‐free media (Figure 1b). With the same inoculum (~0.035 g DW/L, DW = dry weight) in liquid culture, the growth curves of the three transgenic hairy roots were comparable to that of the wild‐type line, all showing a growth cycle of 15–18 days and exhibiting a long exponential growth phase between day 3 and 15 and entering the stationary phase after day 15 (Figure 1c). The harvested root tissue biomass of these cultures was 12.3–13.7 g DW/L, and the specific growth rate (μ) was calculated as 0.30–0.32 day^−1^. This represented a 350‐ to 390‐fold increase in biomass within 18 days of culture. The specific growth rate was even higher than the fast‐growing tobacco BY‐2 cells expressing the (SP)_32_‐EGFP (0.20–0.22 day^−1^) (Zhang *et al*., 2016b).

### Recombinant protein production and secretion in hairy root cultures

Transgenic hairy roots were grown in liquid Schenk and Hildebrandt (SH) medium for 18 days before detection of the recombinant EGFP accumulation within root tissues (estimated by Western blot densitometry, data not shown) and in culture media. As shown in Table 1, both (SP4)_18_ and (SP)_32_ modules dramatically increased the EGFP secretion from the cultured roots, reaching a secreted protein yield of 28.2 ± 3.4 and 24.6 ± 2.5 mg/L respectively. This represented a 56‐fold and 50‐fold increase in secreted protein yield compared with the expression of EGFP control (0.5 ± 0.2 mg/L). The secreted EGFP fused with the (SP4)_18_ or (SP)_32_ module accounted for 88.1% and 95.3% of the total accumulated EGFP protein (secreted and inside root tissues combined) respectively. In contrast, most of the synthesized EGFP control was retained within root tissues, and only 17.9% of which was extracellularly secreted. Due to enhanced protein secretion with the HypGP module, the total recombinant protein (EGFP) yield of the hairy root culture was dramatically increased from 2.8 mg/L to 32.0 mg/L and 25.8 mg/L with the (SP4)_18_ and (SP)_32_ module respectively.

**Table 1:** Recombinant EGFP yields of the hairy root cultures in liquid media for 18 days

Protein expressed†	Media (mg/L)	Roots (mg/gFW)	Total accumulated protein (mg/L)‡	Secreted (%)
(SP4)_18_‐EGFP	28.2 ± 3.4^a^	0.028 ± 0.001^a^	32.0	88.1
(SP)_32_‐EGFP	24.6 ± 2.5^a^	0.009 ± 0.005^b^	25.8	95.3
EGFP	0.5 ± 0.2^b^	0.025 ± 0.004^a^	2.8	17.9

Each value represents the mean of five selected hairy root lines ± SD (*n* = 5). Different letters (a, b) indicate significant difference as determined by a one‐way ANOVA followed by Tukey post hoc range test (*P *<* *0.05).

† The EGFP *equivalent* of the (SP)_32_‐EGFP and (SP4)_18_‐EGFP fusion proteins was quantified.

‡ The proteins accumulated in both root tissues and culture media.

Anti‐EGFP Western blotting detected the *EGFP*,* (SP4)*_*18*_ *‐EGFP* and *(SP)*_*32*_ *‐EGFP* transgene products accumulated in culture media and within root tissues (Figure 1d). The engineered (SP4)_18_ or (SP)_32_ module substantially increased the molecular size of fused EGFP from 27 kDa to more than 100 kDa. For the expression of (SP4)_18_‐EGFP, two distinct bands (~68 kDa lower band and ~120 kDa upper band) were detected within the root tissues, but the upper band dominated in the culture media (>95% estimated by densitometry). In contrast, the *(SP)*_*32*_ *‐EGFP* transgene products were completely segregated between the root tissues and culture media. While the products within root tissues appeared as two bands at 40 and 42 kDa, the secreted product migrated as a broad band at ~115 kDa. As shown below, the secreted (SP4)_18_‐EGFP and (SP)_32_‐EGFP proteins were later determined as being normally Hyp‐*O*‐glycosylated.

Interestingly, when the transgenic hairy roots were grown in Murashige and Skoog (MS) medium, which contains 2.2‐fold more nitrogen (60.1 mm) than SH medium (27.3 mm) (Zhang *et al*., 2016a), a difference in transgene product secretion was observed compared with the cultures in SH medium (Figures S3 and 1d). While significant amount of the (SP4)_18_‐EGFP product (120 kDa) was still secreted into culture medium (8.5 ± 2.6 mg/L, *n* = 3), hardly any secreted EGFP control and (SP)_32_‐EGFP product was detected (Figure S3). However, the transgene products accumulated within root tissues, including the distinct 68 and 120 kDa (SP4)_18_‐EGFP products and the 40–42 kDa (SP)_32_‐EGFP products, were similar between the cultures in MS and SH medium, as estimated by their relative band densities to the EGFP standard based on densitometry assay (Figures 1d and S3). It seemed that the Hyp‐*O*‐glycosylation and subsequent secretion of the AGP‐styled (SP)_32_ module was substantially affected by the medium composition, but this was not observed for the extensin‐styled (SP4)_18_ module.

### Kinetics of recombinant protein synthesis and accumulation in hairy root cultures

**Figure 2:**
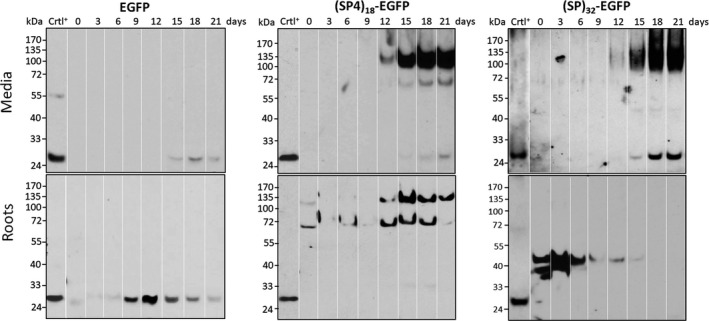
Time course of accumulation of recombinant EGFP, (SP4)_18_‐EGFP and (SP)_32_‐EGFP in culture media and inside root tissues during the hairy root cultures for 21 days. The transgene products were detected by anti‐EGFP Western blotting. The lanes in each panel correspond to the samples harvested at different culture time (from 0 to 21 days). Fifteen μL of culture media or 10 μL clarified root extracts was loaded into each well. Crtl^+^: EGFP standard (50 ng).

The time course of EGFP accumulation in the root tissues and secreted into media of the hairy roots grown in SH medium were further characterized. As shown in Figure 2, dramatic accumulation of the (SP4)_18_‐ or (SP)_32_‐tagged EGFP in culture media was found after 12 days of culture, which correlated with a rapid increase in root biomass as shown in Figure 1c. In contrast, trace amount of EGFP control was secreted into culture media over the growth cycle. While the majority of secreted (SP4)_18_‐EGFP products was Hyp‐*O*‐glycosylated (identified below) with an approximate molecular size of 120 kDa, two distinct products (120 kDa upper band and 68 kDa lower band) co‐occurred within the root tissues, and their ratios changed over the growth cycle, from a low of 20%–30% at early stage (day 0–6) to a high of 80%–90% at late stage (day 15–21). In contrast, the *(SP)*_*32*_ *‐EGFP* transgene products were completely segregated between root tissues and culture media over the growth cycle. The secreted (SP)_32_‐EGFP, detectable from day 12, appeared as an extensively Hyp‐*O*‐glycosylated product with a smear band (averaged at ~115 kDa) reflecting the addition with heterologous polysaccharides (identified below). The products inside roots, migrating as two bands at 40 and 42 kDa, accumulated in considerable amounts at early stage (day 0–3) but substantially decreased during exponential growth (day 6–16), and then became almost undetectable at the end of the culture, presumably due to rapid secretion.

### Subcellular localization of expressed proteins

**Figure 3:**
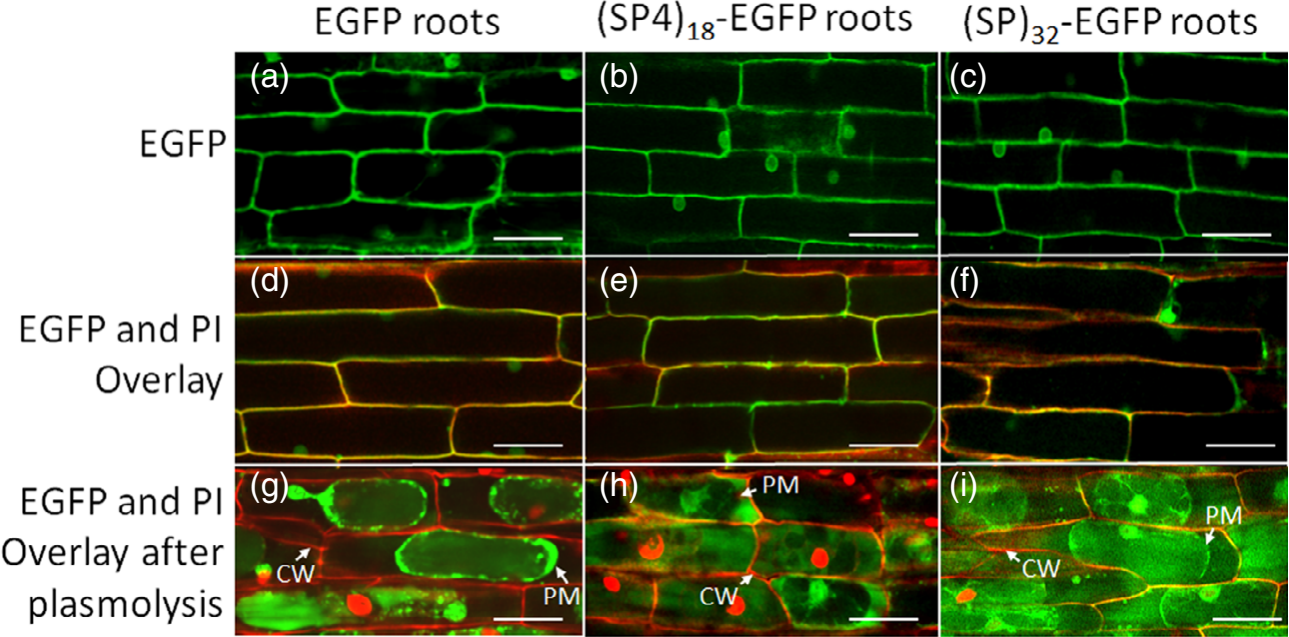
Fluorescence micrographs of hairy roots expressing EGFP, (SP4)_18_‐EGFP and (SP)_32_‐EGFP. The hairy roots grown in petri dish for 12 days were inspected using a laser‐scanning confocal microscope with a 40× water‐immersion objective. The cell wall of the root tissues was stained with propidium iodide (PI). (a–c) Hairy root images detected under green fluorescence channel (488 nm excitation with 525/50 nm filter); (d–f) Overlaid images captured under both green fluorescence channel and red fluorescence channel (543 nm excitation with 595/50 nm filter); (g–i) Hairy root cells plasmolysed with 800 mm mannitol. The images were then detected under both green fluorescence and red fluorescence channels and overlaid. CW, cell wall; PM, plasma membrane. Scale bar = 50 μm.

The green fluorescence of the transgene products in hairy roots was examined under confocal microscope (Figure 3). The hairy roots expressing all the three EGFP gene constructs showed fluorescence at cell wall/plasma membrane interface and around nucleus (Figure 3a–f), similar to that reported earlier (Pham *et al*., 2012). The details for overlay of the hairy root images captured in green fluorescence channel (for EGFP) and red fluorescence channel (for propidium iodide‐stained cell wall) (Figure 3d–f) are shown in Figure S4. When the root tissues were plasmolysed in 800 mm mannitol, the green fluorescence of expressed EGFP control was retained intracellularly despite having the secretion signal peptide (Figure 3g). In contrast, the root cells expressing (SP4)_18_‐ or (SP)_32_‐tagged EGFP showed intense green fluorescence between the plasma membrane and the cell wall (Figure 3h,i), likely in transit to extracellular space, though some intracellular EGFP, particularly those around nucleus, was apparent. This correlated with the detection of considerable amounts of secreted (SP4)_18_‐EGFP or (SP)_32_‐EGFP and trace amount of EGFP in the culture media (Figure 2).

### Structure characterization of the transgene products secreted from hairy roots

**Figure 4:**
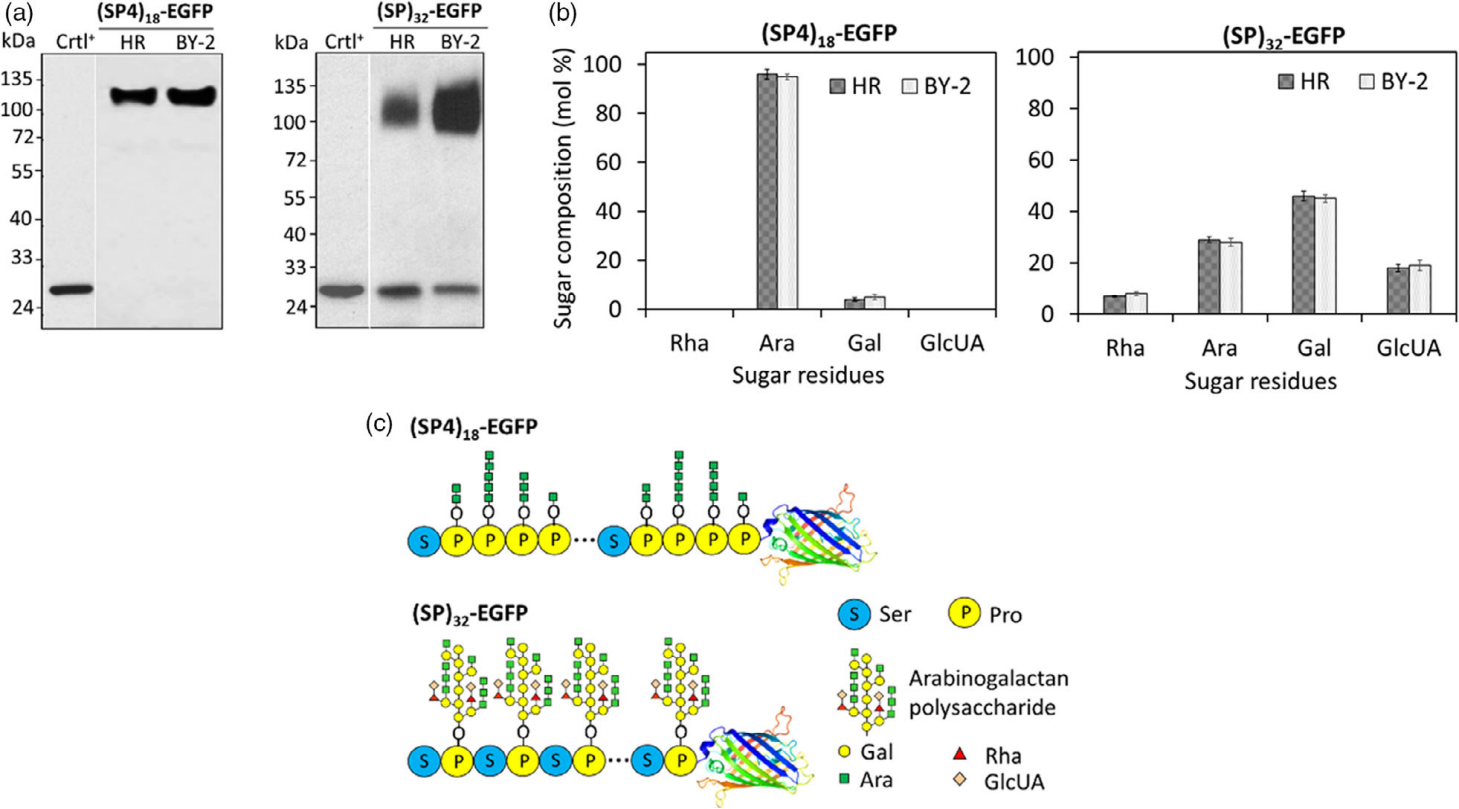
Characterization of the secreted *(SP4)*_*18*_ *‐EGFP* and *(SP)*_*32*_ *‐EGFP* transgene products harvested from hairy root cultures. (a) Anti‐EGFP Western blotting detection of the (SP4)_18_‐EGFP and (SP)_32_‐EGFP proteins secreted from hairy root and BY‐2 cell cultures. The culture media harvested from BY‐2 cell culture were diluted by five times before the assay. Fifteen μL of culture media was loaded into each well. Crtl^+^: EGFP standard (50 ng). (b) Monosaccharide composition of the purified (SP4)_18_‐EGFP and (SP)_32_‐EGFP. The molar percentage (mol %) represents the mean of three measurements ± standard deviation. (c) Molecular structure of Hyp‐*O*‐glycosylated (SP4)_18_‐EGFP and (SP)_32_‐EGFP proteins secreted by tobacco hairy roots or BY‐2 cells, as derived from early characterization of these two proteins produced in BY‐2 cell culture (Shpak *et al*., 1999, 2001; Tan *et al*., 2010; Xu *et al*., 2007, 2008).

The amino acid composition of the secreted (SP)_32_‐EGFP or (SP4)_18_‐EGFP products closely resembled that calculated based on their cDNA sequence, and a high percentage of Hyp residue was detected for both the products (Table S2). When the secreted (SP)_32_‐EGFP or (SP4)_18_‐EGFP glycoproteins were run side‐by‐side with those secreted from BY‐2 cell culture on a SDS‐PAGE gel, they migrated as bands of the same size and appearance (Figure 4a). Sugar assay of the purified (SP)_32_‐EGFP and (SP4)_18_‐EGFP indicated that these proteins were extensively glycosylated with sugar accounting for 41.3 ± 0.5% (w/w) and 60.8 ± 0.9% (w/w) of the (SP4)_18_‐EGFP and (SP)_32_‐EGFP respectively (*n* = 3), which were comparable to their counterpart secreted in BY‐2 cell cultures (Shpak *et al*., 1999, 2001; Xu *et al*., 2008; Zhang *et al*., 2016b), so were the monosaccharide compositions. As shown in Figure 4b, arabinose (Ara) was the dominant sugar residue of (SP4)_18_‐EGFP, accounting for 96 mol% of the total sugar attached with the rest 4 mol% being galactose (Gal). In contrast, the glycans attached to (SP)_32_‐EGFP were rich in Gal (46 mol%) and Ara (30 mol%) with lesser amounts of rhamnose (Rha, 7 mol%) and glucuronic acid (GlcUA, 17 mol%). These results indicated the secreted (SP)_32_‐EGFP and (SP4)_18_‐EGFP in tobacco hairy root culture were the same as those produced by BY‐2 cells whose molecular structures were well characterized (Shpak *et al*., 1999, 2001; Xu *et al*., 2008) and displayed in Figure 4c.

### Structure characterization of transgene products accumulated within root tissues

**Figure 5:**
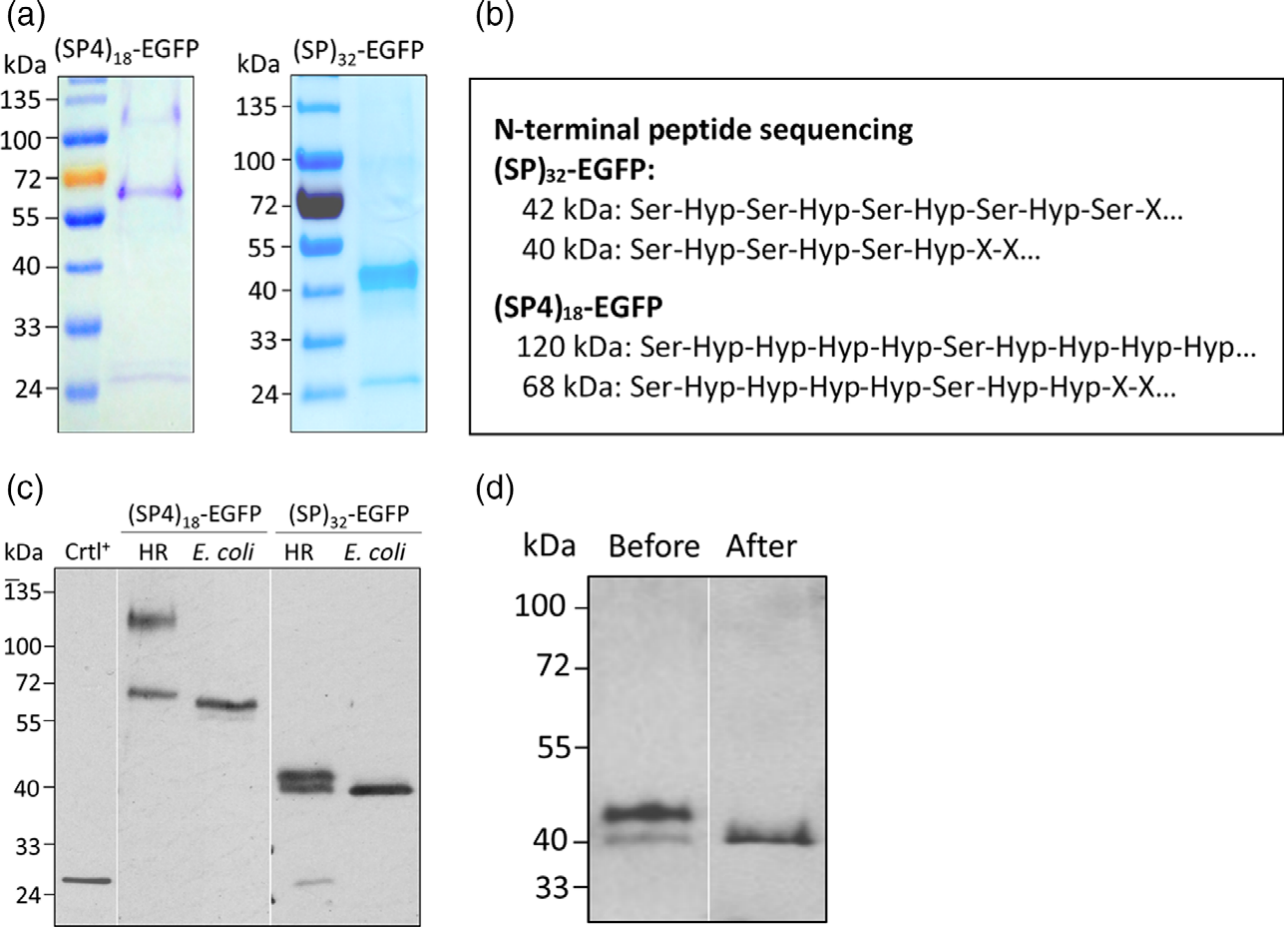
Identification of the *(SP4)*_*18*_ *‐EGFP* and *(SP)*_*32*_ *‐EGFP* transgene products accumulated in root tissues. (a) SDS‐PAGE separation of (SP4)_18_‐EGFP and (SP)_32_‐EGFP products purified from the root tissues. Five μg each of the protein samples was run on a 4%–20% Tris‐HCl gel and stained with Coomassie blue; (b) N‐terminal peptide sequencing of the purified transgene products. ‘X’ denotes unidentified amino acid; (c) Anti‐EGFP Western blotting assay of the (SP4)_18_‐EGFP or (SP)_32_‐EGFP expression in hairy roots (HR) and *E. coli*. Ten μL of clarified root extracts (12‐day‐old culture) or 5 μL of *E. coli* lysates was loaded. Crtl^+^: EGFP standard (50 ng). (d) Anti‐EGFP Western blotting detection of the (SP)_32_‐EGFP products within root tissues before and after β‐elimination reaction. Ten μL of clarified root extract (before treatment) or root extract equivalent (after treatment) was loaded.

Interestingly, the molecular sizes of some *(SP4)*_*18*_ *‐EGFP* product (68 kDa) and all the *(SP)*_*32*_ *‐EGFP* products (40–42 kDa) accumulated within root tissues were greatly different from those secreted into media (Figures 1d and 2). Identification of these plausible intermediate products would help understand the Hyp‐*O*‐glycosylation process of HRGPs. With GFP‐Trap^®^ immunoprecipitation, the roots‐accumulated (SP4)_18_‐EGFP and (SP)_32_‐EGFP products were purified and confirmed by resolving on SDS‐PAGE gels (Figure 5a).

Amino acid composition assay (Table S2) and N‐terminal peptide sequencing (Figure 5b) of the two *(SP4)*_*18*_ *‐EGFP* transgene products (68 and 120 kDa) confirmed that both were (SP4)_18_‐EGFP products with a high percentage of Hyp residue and all the Pro residues in the sequenced region were hydroxylated. The presence of two distinct molecules must result from differential posttranslational modifications on their common polypeptide backbone. The upper band, migrating at the same size as that secreted into media, was obviously the oligo‐arabinosylated (SP4)_18_‐EGFP product as shown in Figure 4c (Shpak *et al*., 2001). The lower band with an apparent molecular size of 68 kDa likely underwent glycosylation too because the molecular size of the non‐glycosylated (SP4)_18_‐EGFP polypeptide was calculated as only 36.9 kDa (Table S1). However, no sugar residue was detected by the alditol acetate method. The *(SP4)*_*18*_ *‐EGFP* gene was subsequently expressed in *E. coli* to generate the non‐glycosylated (SP4)_18_‐EGFP polypeptide (Appendix S3). Surprisingly, the *E. coli*‐produced (SP4)_18_‐EGFP appeared only slightly smaller than the hairy roots‐produced counterpart (1–2 kDa smaller due to lack of Pro hydroxylation) (Figure 5c). This revealed that the 68 kDa molecule was actually the non‐glycosylated (SP4)_18_‐EGFP, and the substantial shift of its apparent molecular size from 36.9 to 68 kDa resulted from its unique polypeptide sequence comprised of a long and very rigid repetitive ‘Ser‐Hyp‐Hyp‐Hyp‐Hyp’ motif, which altered its migration on a SDS‐PAGE gel. In fact, the oligo‐arabinosylated (SP4)_18_‐EGFP (upper band) also substantially shifted its molecular size from the calculated 66 kDa (Table S1) to ~120 kDa.

Amino acid composition assay (Table S2) and *N*‐terminal peptide sequencing (Figure 5b) also confirmed the two *(SP)*_*32*_ *‐EGFP* transgene products (40 and 42 kDa) consisted of (SP)_32_‐EGFP polypeptide with a high percentage of Hyp residue and all the Pro residues in the sequenced region were hydroxylated. Because the apparent molecular size of these two molecules was significantly larger than their polypeptide backbone calculated as 33.4 kDa (Table S1), they were initially regarded as being partially Hyp‐*O*‐glycosylated. When the (SP)_32_‐EGFP polypeptide was expressed in *E. coli* (Appendix S3), it migrated almost the same size as the 40 kDa product on a SDS‐PAGE gel (Figure 5c), indicating the 40 kDa product was the non‐glycosylated (SP)_32_‐EGFP. Again, the shift of apparent molecular size resulted from the rigid repetitive ‘Ser‐Pro’ peptide sequence (but not as rigid as the Ser‐Hyp‐Hyp‐Hyp‐Hyp motifs). Then, the 42 kDa product must be the (SP)_32_‐EGFP polypeptide with further glycosylation either on the Hyp or Ser residue. The product was subject to β‐elimination treatment known to remove sugars *O*‐linked to Ser/Thr (Li *et al*., 2003). As seen in Figure 5d, the 42 kDa band disappeared, whereas intensity of the 40 kDa band increased after the treatment. In addition, when a new synthetic AGP module consisting of 20 repeats of ‘Ala‐Pro’ motif or (AP)_20_ was expressed in tobacco plants as fusion with EGFP, only one single band of product (~38 kDa) was consistently detected in leaves (Figure S5) due to absence of glycosylation on Ala residue. These results indicated the 42 kDa band corresponds to the (SP)_32_‐EGFP polypeptide backbone with further modification on Ser residues, most likely *O*‐galactosylation with a single galactose. Ser‐*O*‐monogalactosylation is a plant‐specific modification of proteins that has been found in both extensins and AGPs (Saito *et al*., 2014; Showalter and Basu, 2016a). In fact, the (SP4)_18_‐EGFP products might be Ser‐*O*‐galactosylated, as ~4 mol% Gal was detected in the secreted (SP4)_18_‐EGFP (Figure 4b).

### Effects of brefeldin A treatment on the glycosylation and trafficking of HypGP modules

**Figure 6:**
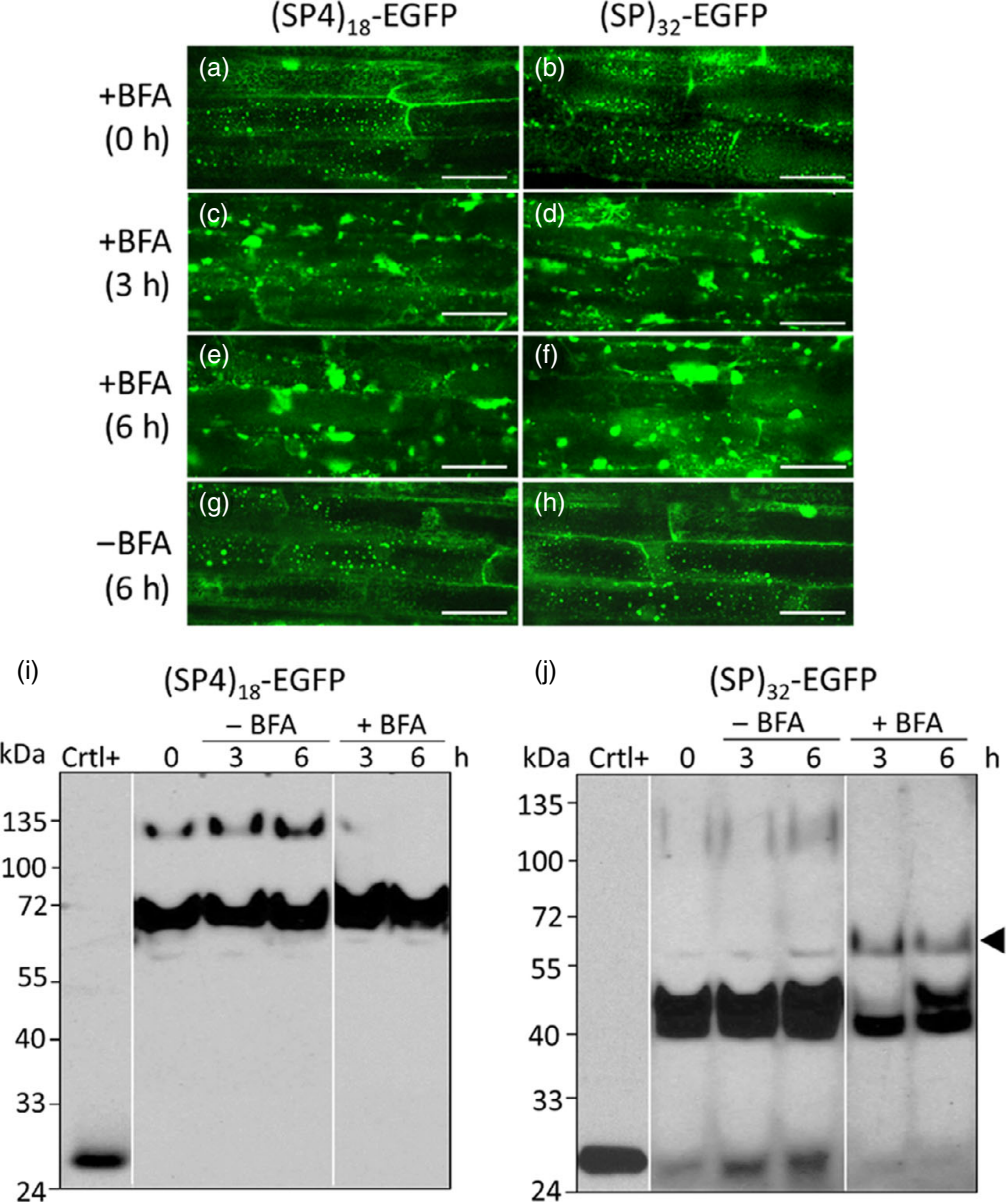
Characterization of the *(SP4)*_*18*_ *‐EGFP* and *(SP)*_*32*_ *‐EGFP* transgene products in the hairy roots treated with BFA. (a–h) Fluorescence micrographs of the cultured hairy roots. Root cells were captured in the cortical cytoplasm under green fluorescence channel (488 nm excitation with 525/50 nm filter) with a 40× water‐immersion objective. A substantial fluorescence signal in a reticulate network throughout the cortical cytoplasm was indicative of ER networks labelled with fluorescent transgene products. Scale bar = 50 μm; (i, j) Western blotting detection of the *(SP4)*_*18*_ *‐EGFP* and *(SP)*_*32*_ *‐EGFP* transgene products, respectively, accumulated in hairy roots. Fifteen μL of clarified root extracts was loaded into each well. Crtl^+^: EGFP standard (50 ng); A new glycoform of (SP)_32_‐EGFP (~60 kDa) formed in the BFA‐treated hairy roots is indicated by the arrow.

To further understand the trafficking and glycosylation of the synthetic (SP4)_18_ and (SP)_32_ modules *in planta*, transgenic hairy roots were treated with the fungal toxin brefeldin A (BFA), which blocks protein transport between the endoplasmic reticulum (ER) and the Golgi apparatus by quickly destroying the Golgi stacks (Ritzenthaler *et al*., 2002). As shown in Figure 6, before the BFA treatment, Golgi stacks were visible as bright fluorescent spots in the root cells (Figure 6a,b), indicating the newly synthesized (SP4)_18_‐EGFP and (SP)_32_‐EGFP molecules were all trafficked through the Golgi apparatus during secretion. After the roots were incubated in BFA‐containing media for 3 and 6 h, there was a progressive reduction in the numbers of free Golgi stacks (fluorescent spots) (Figure 6c–f), just as previously described for several Golgi‐localized GFP fusion proteins expressed in tobacco BY‐2 cells (Ritzenthaler *et al*., 2002; Saint‐Jore‐Dupas *et al*., 2006). In contrast, fluorescent spots were still clearly visible in untreated root cells (Figure 6g,h). Noticeably, massive fluorescent aggregates of irregular shapes measuring 5–15 μm in length occurred in the BFA‐treated root cells (Figure 6c–f). Some of these aggregates, particularly those around the nuclei, were like the perinuclear pleiomorphic aggregates as described earlier in the BFA‐treated BY‐2 cells (Ritzenthaler *et al*., 2002), whereas the others were likely protein bodies formed due to concentrating of the EGFP products in ER, as reported recently for some GFP‐derived proteins overexpressed in tobacco leaves and BY‐2 cells (Hakkinen *et al*., 2018; Saberianfar *et al*., 2015). These changes indicated that BFA treatment successfully blocked the transport of *(SP4)*_*18*_ *‐EGFP* and *(SP)*_*32*_ *‐EGFP* transgene products from ER to Golgi apparatus.

In terms of Hyp‐*O*‐glycosylation of the transgene products in root cells, the glycosylated (SP4)_18_‐EGFP product (120 kDa) disappeared following BFA treatment, whereas the accumulation of the non‐glycosylated product (68 kDa) slightly increased (Figure 6i). This indicated that the glycosylated (SP4)_18_‐EGFP occurred in the Golgi and the non‐glycosylated molecule tended to accumulate in ER under normal growth conditions. Similarly, the non‐glycosylated (SP)_32_‐EGFP product (40 kDa) remained unchanged in the root tissues incubated with BFA, but the plausible Ser‐*O*‐galactosylated product (42 kDa) disappeared 3 h post BFA treatment and then came back at 6 h (Figure 6j). This also indicated the non‐glycosylated (SP)_32_‐EGFP molecule accumulated in the ER, but the 42 kDa molecule resided in Golgi. As the serine‐*O*‐galactosyltransferase was found earlier to be predominantly localized to the ER (Saito *et al*., 2014), the 42 kDa molecule should be generated from Ser‐*O*‐galactosylation of the (SP)_32_‐EGFP polypeptide in ER and then quickly moved to Golgi, where further modification with Hyp‐*O*‐glycosylation occurred (presumably upon induction). The rapid disruption of Golgi stacks with BFA treatment blocked the trafficking of the 42 kD molecule to Golgi, triggering the secretion of this product via routes that are independent of the ER‐Golgi pathway, termed nonclassical or unconventional secretion pathway, to generate small amount of a new (SP)_32_‐EGFP product with a molecular size of ~60 kDa (Figure 6j). Increasing number of published researches in recent years indicated that such unconventional secretions are common to all eukaryotes, including plants (Poulsen *et al*., 2014; Rose and Lee, 2010; Wang *et al*., 2010). Due to reduced BFA effect on protein trafficking 6 h post BFA treatment, the 42 kD molecule reappeared in the root cells.

## Discussion

*In vitro* culture of the fully differentiated plant organ, hairy roots, integrates the merits of plant cell culture with those of whole‐plant cultivation, and it is emerging as a promising bioproduction system for recombinant proteins with therapeutic application. In addressing the low protein productivity challenge, we engineered designer HypGP modules as molecular carriers to boost secreted protein yields. This technology was previously developed in tobacco cell culture, and most recently demonstrated in microalgae culture, to dramatically increase secreted protein yields (Ramos‐Martinez *et al*., 2017; Xu *et al*., 2007, 2010; Zhang *et al*., 2016b). However, the broader application of this technology in differentiated plant organs, such as hairy roots or even whole plants, has yet to be explored. In this study, two types of designer HypGP modules, including (SP4)_18_ comprised of the signature peptide sequence of extensins and (SP)_32_ comprised of the signature peptide sequence of AGPs, were engineered into tobacco hairy roots to evaluate their function in boosting the secreted yields of the fused EGFP protein.

On the other hand, engineering a synthetic extensin or AGP glycosylation module fused to a reporter protein provided an innovative platform for studying the Hyp‐*O*‐glycosylation of plant cell wall glycoproteins, particularly AGPs that are highly heterogeneous and complex in glycosylation (Showalter *et al*., 2010; Tan *et al*., 2012). While the synthetic (SP4)_18_ and (SP)_32_ modules were predicted to undergo extensive Hyp‐*O*‐glycosylation *in planta* as endogenous extensins and AGPs, the fused reporter protein facilitated detection, purification and subcellular localization of the expressed glycomodules. In addition, various vesicular transport inhibitors, such as BFA, can be easily introduced into the *in vitro* culture system to further delineate the important steps in trafficking and Hyp‐*O*‐glycosylation of the engineered HypGP modules.

The engineered (SP4)_18_ or (SP)_32_ module substantially enhanced the secretion of EGFP. Secreted protein yields achieved in this study represented one of the highest among tobacco hairy root cultures, where protein yields of less 5.0 mg/L were typically reported (Georgiev *et al*., 2012; Hakkinen *et al*., 2014; Pham *et al*., 2012). However, compared with the BY‐2 cell culture expressing (SP)_32_‐EGFP (up to 131 mg/L) (Zhang *et al*., 2016b), the secreted protein yield obtained in this study was still low, which might be attributed to the diffusion limited through the apoplastic pathway to the tissue exterior. This was reflected in the fluorescent micrographs of the root cells, in which significant amount of green fluorescence was still detected (Figures 3 and 6), while in contrast much less green fluorescence was observed inside BY‐2 cells (Zhang *et al*., 2016b). Similar findings were reported by Hakkinen *et al*. (2014) who compared the expression of a recombinant antibody M12 in different plant expression platforms (leaves, hairy roots, BY‐2 cells and moss), and found that BY‐2 cell suspension culture produced the highest overall secreted antibody yields.

An extremely high secreted yields of GFP (120 mg/L) was recently reported with a turnip (*Brassica rapa rapa*) hairy root culture (Huet *et al*., 2014). However, when the same expression vector was transformed into tobacco (*N. tabacum* cv. SR1) hairy roots, GFP was present in the medium at levels over two orders of magnitude (only 0.77 mg/L) less than in turnip root culture (Huet *et al*., 2014), comparable to that obtained in this study (expression of the EGFP control). In another report, the secreted yields of *E. coli* B‐subunit heat‐labile toxin antigen produced in *Petunia* and tobacco hairy roots were approximately seven times higher than in tomato (De Guzman *et al*., 2011). This suggests a high variability in the efficiency of various plant species for expression and secretion of recombinant proteins. Due to ease of transformation, excellent growth characteristics and low extracellular protease activity, tobacco has been regarded as a preferred species for production of heterologous proteins (Xu *et al*., 2012).

As the Hyp‐*O*‐glycosylation ‘code’ predicts, the engineered synthetic HypGP peptides underwent Hyp‐*O*‐glycosylation in tobacco hairy roots. In fact, our results demonstrated that the extensive Hyp‐*O*‐glycosylation of the HypGP modules, either with arabino‐oligosaccharides on the (SP4)_18_ module or arabinogalactan polysaccharides on the (SP)_32_ module, was essential in facilitating secretion of the fused protein (EGFP), because almost all the protein products harvested in media, including the120 kDa (SP4)_18_‐EGFP and 115 kDa (SP)_32_‐EGFP products, were Hyp‐*O*‐glycosylated. In contrast, virtually all the EGFP control and the non‐glycosylated (SP4)_18_‐EGFP and (SP)_32_‐EGFP products were retained in root tissues. When the entire population of the *(SP4)*_*18*_ *‐EGFP* or *(SP)*_*32*_ *‐EGFP* transgene products were examined, they appeared totally different in size between those accumulated in root tissues and secreted into media. This was unexpected, but it provided a valuable system to study the Hyp‐*O*‐glycosylation process for HRGPs, because the difference in molecular sizes of the transgene products must result from the differential glycosylation on a common polypeptide backbone.

Many previous work studying the biosynthesis of HRGPs and their trafficking in plant cells speculated a model in which hydroxylation of Pro residues occurs in ER and subsequent construction of the glycan structure occurs by sequential addition of sugar residues in the Golgi apparatus (Kieliszewski, 2001; Showalter and Basu, 2016a; Velasquez *et al*., 2012), though the prolyl 4‐hydroxylase and certain glycosyltransferases were found to locate in both ER and Golgi apparatus (Basu *et al*., 2013; Oka *et al*., 2010; Yuasa *et al*., 2005). The detection of distinct hydroxylated but non‐Hyp‐*O*‐glycosylated transgene products in root cells, either the 68 kDa (SP4)_18_‐EGFP or the 40/42 kDa (SP)_32_‐EGFP (Figures 1d, 2 and 6b) provided direct evidence supporting this model. It also indicated a rapid and constitutive process for the hydroxylation of the HypGP modules in the ER because all the Pro residues in the peptide‐sequenced region were hydroxylated (Figure 5b). However, the accumulation of significant amounts of non‐Hyp‐*O*‐glycosylated intermediates inside root cells indicated that the protein trafficking or addition of glycans to the Hyp residues may comprise a rate‐limiting step for extensins and AGPs.

**Figure 7:**
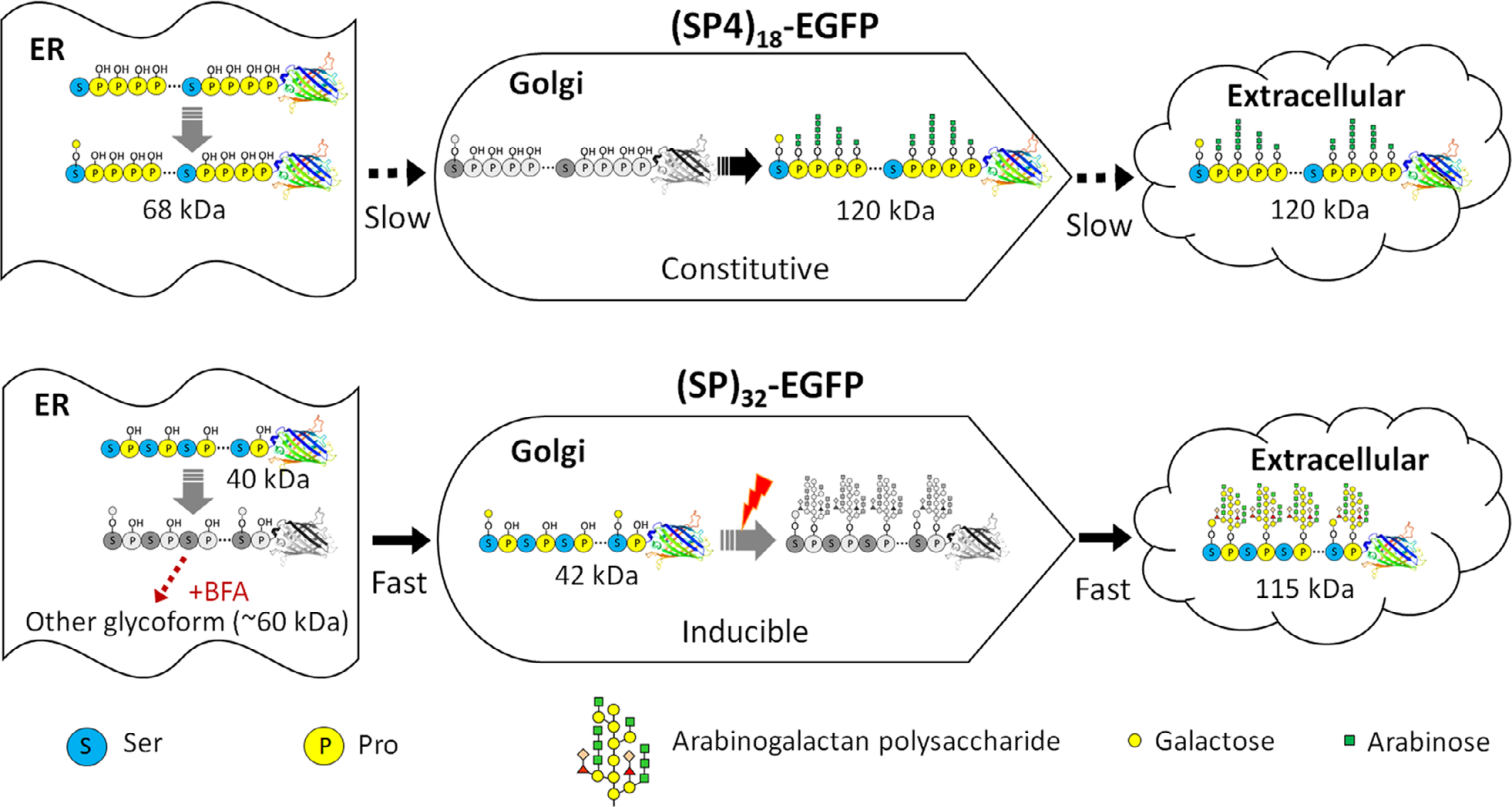
Schematic representation of the proposed model for trafficking, glycosylation and extracellular secretion of engineered HypGP modules in hairy roots. The molecular structures in colour describe the molecules accumulating in secretory pathway, being detectable by Western blotting; the molecular structures in black and white describe the molecules occurring in secretory pathway without accumulation (undetectable by Western blotting).

Hyp‐*O*‐glycosylation of the (SP4)_18_ module, which represents the posttranslational modifications of plant extensins, involves addition of 1–5 arabinose residues to the contiguous Hyp residues (Rautengarten *et al*., 2017; Shpak *et al*., 2001). At least eight arabinosyltransferases involved in the glycosylation have been identified in *Arabidopsis* (Showalter and Basu, 2016a). Hyp‐*O*‐glycosylation of the (SP)_32_ module, which represents the posttranslational modifications of plant AGPs, is more complicated and involves addition of large and branched arabinogalactan polysaccharide chains to the non‐contiguous Hyp residues. To date, 17 different genes corresponding to seven distinct enzymes involved with AGPs glycosylation have been identified (Showalter and Basu, 2016a). However, the precise Hyp‐*O*‐glycosylation process of either extensins or AGPs has yet been understood as far. The key observations made in this study prompted us to propose an updated model regarding the trafficking, glycosylation, and extracellular secretion of the (SP4)_18_ and (SP)_32_ modules, or extensins and AGPs in general, as depicted in Figure 7. In general, all the Pro residues in the engineered (SP4)_18_ and (SP)_32_ modules were rapidly hydroxylated to be Hyp in ER, where *O*‐galactosylation also occurred on a small percentage of Ser residues. The (SP4)_18_ and (SP)_32_ modules were transported to Golgi for further modifications on Hyp residues with *O*‐linked glycans, either arabino‐oligosaccharides or arabinogalactan polysaccharides depending on the polypeptide sequence. The Hyp‐*O*‐glycosylated molecules were secreted into extracellular space at different rates between the (SP4)_18_ and (SP)_32_ modules.

For the extensin‐styled (SP4)_18_ module, the trafficking of the non‐Hyp‐*O*‐glycosylated molecule (68 kDa) from ER to Golgi was rate‐limited, leaving the accumulation of these molecules in ER without being affected by the BFA treatment (Figure 6i). The Hyp‐*O*‐arabinosylation of the (SP4)_18_ module in Golgi was a constitutive process, allowing consistent detection of the glycosylated molecule in root tissues (grown in both SH and MS medium) and transgenic plantlets (Figures 1d, 2, S1 and S3). In addition, the extracellular secretion of the glycosylated (SP4)_18_‐EGFP was a rate‐limiting process, leading to intracellular accumulation of significant amounts of this product that co‐occurred with the non‐glycosylated molecule (Figures 1d and 2).

For the AGP‐styled (SP)_32_ module, while the non‐glycosylated polypeptide (40 kDa) tended to accumulate in ER, the trafficking of the Ser‐*O*‐glycosylated (SP)_32_‐EGFP molecule (42 kDa) from ER to Golgi is a rapid process, leaving accumulation of this molecule in the Golgi. Upon BFA treatment to destroy the Golgi apparatus, the 42 kDa molecule quickly disappeared but the 40 kDa molecule remained unchanged (Figure 6j). Unlike the (SP4)_18_ module, the Hyp‐*O*‐arabinogalactosylation of the (SP)_32_ module is an inducible process, presumably triggered by stresses, for example nitrogen deficiency in SH medium (Figure 1d) or drought imposed to the transgenic plants (Figure S6). However, the extracellular secretion of the glycosylated (SP)_32_‐EGFP is a rapid process, hardly any glycosylated (SP)_32_‐EGFP product (115 kDa) was detected inside root tissues (Figures 1d and 2). Therefore, the (SP)_32_ module, heavily *O*‐glycosylated with arabinogalactan polysaccharides, may function more efficiently than the oligo‐arabinosylated (SP4)_18_ module in facilitating secretion of the fused protein. Our data also have implications for elaborating the arabinogalactan glycans. During the entire culture cycle, no intermediate (SP)_32_‐EGFP product between 42 and 115 kDa was detected (Figure 2), suggesting that either this process was very efficient, or a pre‐made sugar complex was added in a single step rather than sequential *de novo* assembly. As Gal is the first sugar residue transferred to the AGP polypeptide backbone to form β‐1,3‐galactan chain (Nguema‐Ona *et al*., 2014; Xu *et al*., 2007) and due to identification of certain Hyp:galactosyltransferases localized to the ER of suspension‐cultured *Arabidopsis* cells (Basu *et al*., 2013; Liang *et al*., 2010; Oka *et al*., 2010), an updated model for AGPs glycosylation was recently proposed (Basu *et al*., 2013; Oka *et al*., 2010; Zhang *et al*., 2016b). Based on this model, the first Gal residue of the Hyp‐glycan is added to peptidyl Hyp in the ER with further glycan chain elongation occurring in the Golgi. Hoverer, this model was somewhat not supported by the findings obtained in this study because no Hyp‐monogalactosylated (SP)_32_‐EGFP product (~5.2 kDa larger than the 42 kDa molecule if present) was detected in root tissues. Continuing research is needed to completely elucidate the Hyp‐*O*‐glycosylation process of AGPs in plants.

## Conclusions

This study is the first report on engineering synthetic HypGP modules in differentiated plant organs–hairy roots and whole plants–to increase recombinant protein expression and characterize Hyp‐*O*‐glycosylation process. Transgenic whole plants only provided explants for generation of hairy roots, whereas this *in vitro* culture system facilitated protein expression studies. Our research demonstrated that designer HypGPs, either the extensin‐styled (SP4)_18_ module or the AGP‐styled (SP)_32_ module, could boost secretion of the fused EGFP by up to 56‐fold. Extensive Hyp‐*O*‐glycosylation of the engineered HypGP modules was essential in facilitating the protein secretion. There were, however, differences in the trafficking, Hyp‐*O*‐glycosylation and extracellular secretion between the engineered (SP4)_18_ and (SP)_32_ modules. The results from this research improves our current understanding regarding the Hyp‐*O*‐glycosylation and extracellular secretion of the cell wall glycoprotein (extensins and AGPs) in plants.

## Experimental procedures

### Expression vectors

Plant expression vectors including *pBI121‐SS* ^*tob*^ *‐EGFP*,* pBI121‐SS* ^*tob*^ *‐(SP)*_*32*_ *‐EGFP* and *pBI121‐SS* ^*tob*^ *‐(SP4)*_*18*_ *‐EGFP* encoding EGFP control and EGFP with the (SP)_32_ and (SP4)_18_ module respectively (Figure 1a) were obtained from the Kieliszewski lab at Ohio University. *SS* ^*tob*^ denotes the tobacco extensin signal sequence.

### Generation of transgenic hairy roots

Transgenic tobacco (*Nicotiana tabacum*) plantlets expressing EGFP, (SP)_32_‐EGFP or (SP4)_18_‐EGFP were created using the *Agrobacterium‐*mediated leaf‐disc method as described in Appendix S1. Transgenic hairy roots were then induced by infecting young leaves of the selected transformants with *Agrobacterium rhizogenes* ATCC 15834. Hairy roots were also induced from wild‐type *N. tabacum*. The obtained hairy roots were maintained in solid SH medium. Hairy root liquid culture and growth rate determination were described in Appendix S2.

### Protein extraction and Western blotting analysis

The collected culture media were directly used for Western blotting assay. For extracting proteins accumulated inside roots, frozen root tissues (0.5 g) were ground by mortar and pestle in liquid nitrogen, and then supplemented with SDS (sodium dodecyl sulphate) extraction buffer (Zhang *et al*., 2016b) at a ratio of 1 : 2 (w/v). Samples were centrifuged at 13 000 × *g* for 15 min and the supernatants were collected for Western blotting assay as described in Appendix S4 using rabbit anti‐EGFP antibody (ThermoFisher Scientific, Waltham, MA).

### Brefeldin A (BFA) treatment of cultured hairy roots

Transgenic hairy roots expressing (SP4)_18_‐EGFP or (SP)_32_‐EGFP were cultured in liquid medium for 8–10 days to reach mid‐exponential growth phase. The cultures were then supplemented with BFA to a final concentration of 50 μg/mL as described early (Saint‐Jore‐Dupas *et al*., 2006), and incubated at room temperature for 3 and 6 h before the root tissues were harvested for confocal microscopic and Western blotting assay.

### Quantification of recombinant proteins

Secreted EGFP or EGFP *equivalent* of recombinant (SP)_32_‐EGFP and (SP4)_18_‐EGFP was quantified by determining the Relative Fluorescence Unit (RFU) of the culture media on a Modulus™ Single Tube Multimode Reader with a Modulus™ Blue Fluorescence Optical Kit (Turner BioSystems Inc., Sunnyvale, CA) (Zhang *et al*., 2016b). EGFP or EGFP *equivalent* accumulated within root tissues was quantified by densitometry based on anti‐EGFP Western blotting. Briefly, samples and EGFP standard were electrophoresed on the same SDS‐PAGE gel. After immunoblot detection with the anti‐EGFP antibody, the contents of EGFP were estimated by comparison of the band intensities on a VersaDoc 4000 imaging system and analysed using the Quant‐1 software (Bio‐Rad, Hercules, CA).

### Laser‐scanning confocal imaging

Confocal imaging of the hairy roots expressing EGFP with or without a HypGP tag was performed using a Nikon D‐Eclipse C1 laser‐scanning confocal head mounted on a Nikon Eclipse E800 microscope with a 40x/0.8 W Nikon Fluor water‐immersion objective. The samples’ fluorescence was excited and detected at the following wavelengths: 488 nm with a 525/50 nm filter for EGFP fluorescence, and 543 nm with a 595/50 nm filter for propidium iodine (PI) fluorescence. For subcellular localization of the expressed EGFP products, the root tissues were plasmolysed with 800 mm mannitol for 3 h before the images were captured using the confocal microscopy.

### Purification of recombinant (SP)_32_‐EGFP and (SP4)_18_‐EGFP

The secreted recombinant proteins were separated from the culture media by hydrophobic interaction chromatography and reverse‐phased HPLC as previously described (Xu *et al*., 2007, 2010). For purification of the proteins from roots, harvested root tissues were ground in liquid nitrogen and pre‐separated with 30% and 60% (w/v) ammonium sulphate precipitation. The fraction precipitated with 60% (w/v) ammonium sulphate was further purified with GFP‐Trap^®^‐agarose beads coupled with GFP binding protein (ChromoTek GmbH, Germany) according to the manufacturers’ procedures.

### N‐terminal peptide sequencing

The purified fusion proteins were separated on a 4%–20% Tris‐HCl gel and stained with Coomassie blue R‐250. The target bands were cut from the gel for peptide sequencing by Edman degradation at the Protein Facility of the Iowa State University (Ames, IA).

### Monosaccharide composition assay

Monosaccharide composition of the purified transgene products was analysed as alditol acetates derivatives by gas chromatography (Brunton *et al*., 2007) on a Shimadzu GC‐2010 equipped with a Rtx^®^‐225 column (Crossbond^®^ 50% cyanopropylmethyl/50% phenylmethyl polysiloxane, 30 m × 0.25 mm × 0.25 μm, Restek, Bellefonte, PA), as described earlier (Ge *et al*., 2012). Uronic acids were assayed by the colorimetric method based on reaction with m‐hydroxydiphenyl, with D‐glucuronic acid as the standard (Blumenkrantz and Asboe‐Hansen, 1973).

### ß‐elimination

(SP)_32_‐EGFP products (~0.5 mg) in root extract was subject ß‐elimination with the GlycoProfile™ ß‐Elimination kit (Sigma, St Louis, MO) at 4 °C for 18 h in accordance with manufacturer's procedures. The sample before and after ß‐elimination was then assayed by anti‐EGFP Western blotting.

### Statistical analysis

Assays of root biomass and recombinant protein yields were carried out with three to five replicates (as indicated in text) and data are presented as the mean with standard deviation (*SD*). One‐way analysis of variance (ANOVA) followed by a Tukey *post hoc* range test was used to determine differences among treatments with *P *<* *0.05 considered to be significant.

## Conflict of interest

The authors declare that they have no competing interests.

## Supporting information


**Figure S1** Anti‐EGFP Western blotting detection of the *EGFP*,* (SP)*_*32*_ *‐EGFP* and *(SP4)*_*18*_ *‐EGFP* transgene products accumulated in the leaves of transgenic plantlets.
**Figure S2** PCR detection of the *rolC*,* aux1* and *virD2* genes using the genomic DNA extracted from transgenic hairy roots as a template.
**Figure S3** Detection of the *EGFP*,* (SP)*_*32*_ *‐EGFP* and *(SP4)*_*18*_ *‐EGFP* transgene products in hairy root cultures in MS medium.
**Figure S4** Confocal laser‐scanning microscopy images of hairy roots expressing EGFP, (SP4)_18_‐EGFP and (SP)_32_‐EGFP.
**Figure S5** Expression of (AP)_20_‐EGFP in tobacco plants and anti‐EGFP Western blotting detection of the transgene product accumulated in tobacco leaves.
**Figure S6** Anti‐EGFP Western blotting detection of the *(SP)*_*32*_ *‐EGFP* transgene products in the leaves of transgenic plants under drought stress.
**Table S1** Comparison of the molecular size of the *EGFP*,* (SP4)*_*18*_ *‐EGFP* and *(SP)*_*32*_ *‐EGFP* transgene products expressed *in planta*.
**Table S2** Amino acid compositions determined for *(SP4)*_*18*_ *‐EGFP* and *(SP)*_*32*_ *‐EGFP* transgene products expressed in hairy roots are compared to those predicted from their cDNA sequence.
**Appendix S1** Generation of stably transformed tobacco plantlets.
**Appendix S2** Hairy root culture and determination of root tissue biomass and growth rate.
**Appendix S3** Expression of (SP)_32_‐EGFP and (SP4)_18_‐EGFP in *E. coli*.
**Appendix S4** SDS‐PAGE and Western blotting assay.
