# Supplementary material for: Engineering ‘designer’ glycomodules for boosting recombinant protein secretion in tobacco hairy root culture and studying hydroxyproline‐O‐glycosylation process in plants
Source: Plant Biotechnol J. 2018 Dec 14;17(6):1130–41. doi: 10.1111/pbi.13043 (PMC6523594; doi:10.1111/pbi.13043)
Supplement: Supplementary file 1 — Figure S1 Anti‐EGFP Western blotting detection of the EGFP, (SP) 32 ‐EGFP and (SP4) 18 ‐EGFP transgene products accumulated in the leaves of transgenic plantlets. Figure S2 PCR detection of the rolC, aux1 and virD2 genes using the genomic DNA extracted from transgenic hairy roots as a template. Figure S3 Detection of the EGFP, (SP) 32 ‐EGFP and (SP4) 18 ‐EGFP transgene products in hairy root cultures in MS medium. Figure S4 Confocal laser‐scanning microscopy images of hairy roots expressing EGFP, (SP4)18‐EGFP and (SP)32‐EGFP. Figure S5 Expression of (AP)20‐EGFP in tobacco plants and anti‐EGFP Western blotting detection of the transgene product accumulated in tobacco leaves. Figure S6 Anti‐EGFP Western blotting detection of the (SP) 32 ‐EGFP transgene products in the leaves of transgenic plants under drought stress. Table S1 Comparison of the molecular size of the EGFP, (SP4) 18 ‐EGFP and (SP) 32 ‐EGFP transgene products expressed in planta. Table S2 Amino acid compositions determined for (SP4) 18 ‐EGFP and (SP) 32 ‐EGFP transgene products expressed in hairy roots are compared to those predicted from their cDNA sequence. Appendix S1 Generation of stably transformed tobacco plantlets. Appendix S2 Hairy root culture and determination of root tissue biomass and growth rate. Appendix S3 Expression of (SP)32‐EGFP and (SP4)18‐EGFP in E. coli. Appendix S4 SDS‐PAGE and Western blotting assay. [file PBI-17-1130-s001.docx]

**Supplementary Materials**

**Table S1. Comparison of the molecular size of the *EGFP*, *(SP4)_18_-EGFP* and *(SP)_32_-EGFP* transgene products expressed *in planta***

| **Gene Construct** | **Theoretical size (kDa)** | | | **Detected size (kDa)** | |
| --- | --- | --- | --- | --- | --- |
|  | Non-glycosylated^†^ | Mono- galactosylated^‡^ | Normally glycosylated^§^ | Intracellular | Secreted |
| *EGFP* | 27.0 | − | − | 27 | 27 |
| *(SP4)_18_-EGFP* | 36.9 | − | ~66 | 68 | 120 |
| *(SP)_32_-EGFP* | 33.4 | 38.6 | ~113 | 40, 42 | 115 |

^†^: Calculated based on hydroxylation of all the Pro residues in the HypGP module.

^‡^: Presumably glycosylated with a single galactose on each Hyp residue, based on the new Hyp-*O*-glycosylation model of AGPs proposed by (Oka et al., 2010). According to this model, the first Gal residue of the Hyp-glycan is added to peptidyl Hyp in ER with further glycan chain elongation occurring in the Golgi.

^§^: Calculated based on addition of an average of 3 arabinose residues on each Hyp in the (SP4)_18_ module (Shpak et al., 2001) and a ~2.5 kDa Hyp-glycan (Tan et al., 2010; Xu et al., 2010) on each Hyp in the (SP)_32_ module.

**Table S2.**  Amino acid compositions determined for *(SP4)_18_-EGFP* and *(SP)_32_-EGFP* transgene products expressed in hairy roots are compared to those predicted from their cDNA sequence^†^

| Amino Acid | Composition (mol %) | | | | | |
| --- | --- | --- | --- | --- | --- | --- |
|  | (SP4)_18_-EGFP | | | (SP)_32_-EGFP | | |
|  | Secreted | Within root tissues^‡^ | cDNA | Secreted | Within root tissues^‡^ | cDNA |
| **Hyp** | **21.5** | **22.0** | **24.8** | **10.8** | **10.4** | **13.8** |
| **Pro** | **3.1** | **3.3** |  | **3.2** | **3.3** |  |
| Asn/Asp | 9.4 | 9.6 | 9.4 | 9.6 | 9.9 | 10.1 |
| Thr | 4.5 | 4.6 | 4.8 | 5.7 | 6 | 5.6 |
| **Ser** | **9.0** | **8.4** | **8.8** | **13.9** | **13.3** | **13.7** |
| Gly | 6.2 | 6.5 | 6.7 | 7.5 | 7.8 | 7.2 |
| Glu/Gln | 7.6 | 7.8 | 7.3 | 7.8 | 7.5 | 7.8 |
| Ala | 2.5 | 2.3 | 2.4 | 2.5 | 2.6 | 2.9 |
| Val | 5.8 | 5.6 | 5.5 | 6.7 | 6.5 | 6.2 |
| Cys | 0.7 | 0.5 | 0.6 | 0.6 | 0.7 | 0.7 |
| Met | 2.2 | 1.9 | 1.8 | 2.5 | 2.3 | 2.0 |
| Ile | 3.3 | 3.5 | 3.6 | 3.3 | 3.6 | 3.9 |
| Leu | 6.6 | 6.8 | 6.4 | 7.5 | 7.3 | 6.9 |
| Tyr | 3.5 | 3.7 | 3.3 | 3.3 | 3.5 | 3.6 |
| Phe | 3.2 | 3.1 | 3.6 | 3.6 | 3.5 | 3.9 |
| His | 2.9 | 2.5 | 2.7 | 2.4 | 2.6 | 2.9 |
| Lys | 5.8 | 6.0 | 6.1 | 7 | 6.9 | 6.5 |
| Arg | 2.2 | 1.9 | 1.8 | 2.1 | 2.3 | 2.0 |
| Trp^§^ | ^_^ | ^_^ | 0.3 | ^_^ | ^_^ | 0.3 |

^†^: The free amino acids were analyzed through precolumn phenylisothiocyanate derivatization and liquid chromatography as described previously (Bergman et al., 1986; Xu et al, 2008).

^‡^: The mixture of 68 kDa/120 kDa (SP4)_18_-EGFP transgene products or 40 kDa/42 kDa (SP)_32_-EGFP transgene products accumulated within root tissues was assayed.

^§^: Not determined


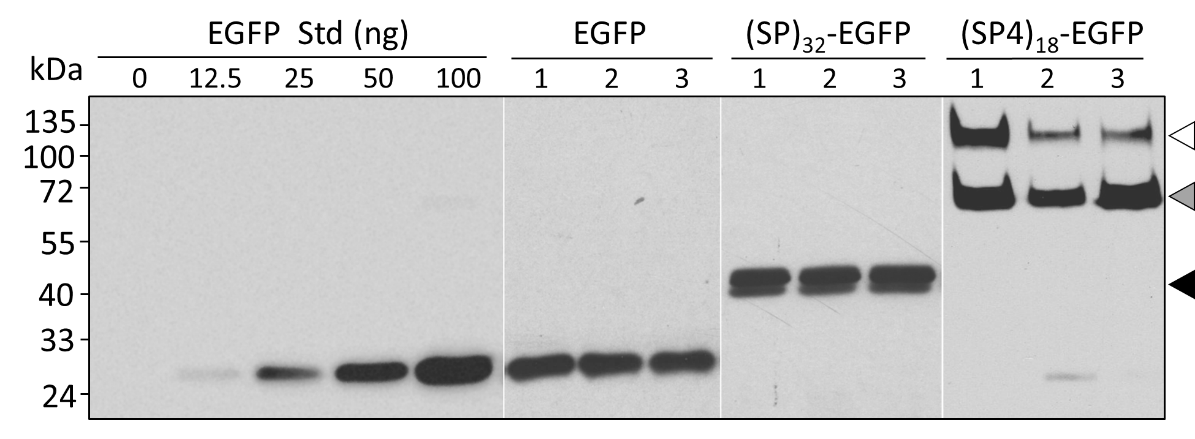


**Figure S1. Anti-EGFP Western blotting detection of the *EGFP*, *(SP)_32_-EGFP* and *(SP4)_18_-EGFP* transgene products accumulated in the leaves of transgenic plantlets**. Three transgenic lines (1, 2 and 3) for each construct were assayed. The tobacco plantlets were grown in Magenta^TM^ box for 2 weeks before the assay. Eight µl of clarified leaf extracts (supernatants) was loaded into each well. The (SP)_32_ module, which was predicted to be Hyp-*O*-glycosylated with arabinogalactan polysaccharides *in planta* and thus dramatically increase the fusion protein size to ~115 kDa (Xu et al., 2008; Zhang et al., 2016), migrated at only 40-42 kDa on the SDS-PAGE gel (indicated by dark arrow). These products were later identified as non-Hyp-*O*-glycosylated (SP)_32_-EGFP in this study. In contrast, extensively Hyp-*O*-glycosylated (SP4)_18_-EGFP fusion protein (120 kDa) was detected in the tobacco leaves (indicated by white arrow), and it co-existed with the non-glycosylated (SP4)_18_-EGFP product (68 kD, indicated by grey arrow) as also identified in this study.


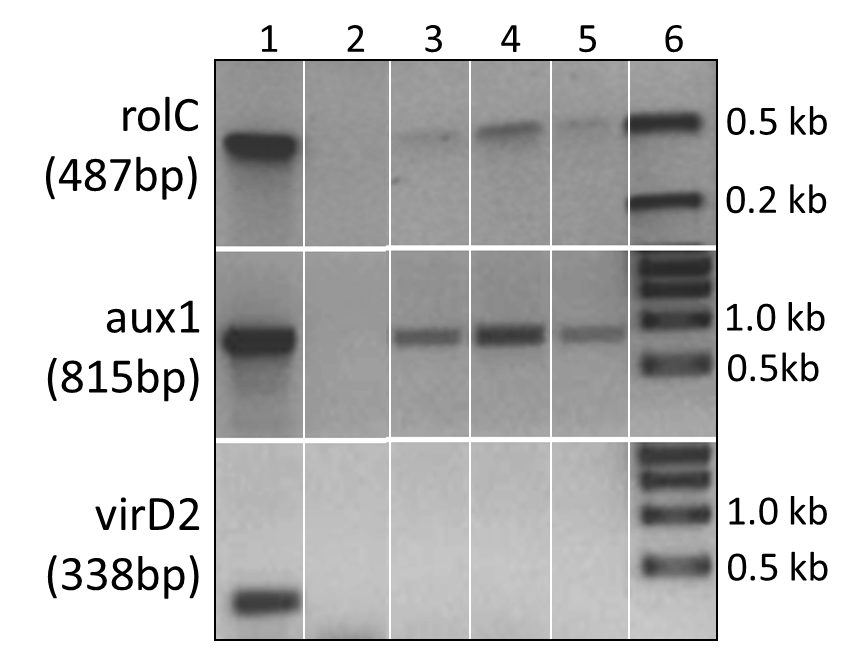


**Figure S2. PCR detection of the *rolC*, *aux1* and *virD2* genes using the genomic DNA extracted from transgenic hairy roots as templates**. Lane 1: positive control (lysate of *A. rhizogenes*, ATCC 15834); Lane 2: negative control (genomic DNA extracted from a transgenic tobacco plant expressing EGFP); Lane 3: hairy roots expressing (SP)_32_-EGFP; Lane 4: hairy roots expressing (SP4)_18_-EGFP; Lane 5: hairy roots expressing EGFP; Lane 6: DNA ladder

**
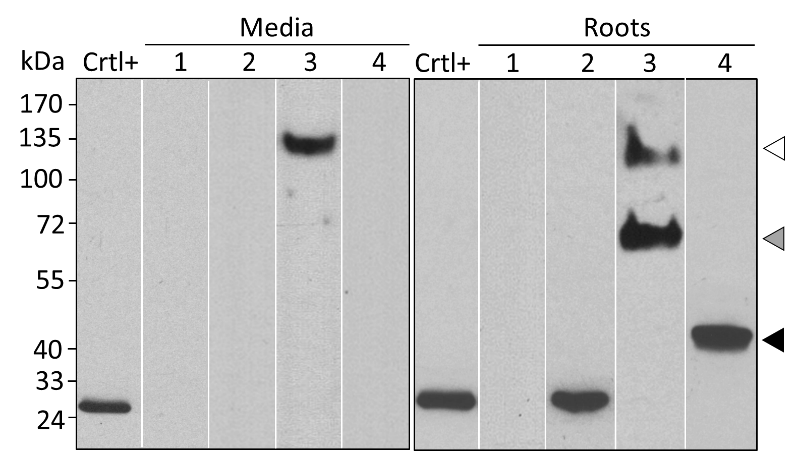
**

**Figure S3.** **Detection of the *EGFP*, *(SP)_32_-EGFP* and *(SP4)_18_-EGFP* transgene products in hairy root cultures in MS medium.** The roots and media were harvested after 12 days of culture for anti-EGFP Western blotting assay. Fifteen µl of culture media or 10 µl of clarified root extracts was loaded in each well. Crtl^+^: EGFP standard (50 ng); Lane 1: Hairy roots induced from a wild type tobacco plant, Lane 2: Hairy roots expressing EGFP control; Lane 3: Hairy roots expressing (SP4)_18_-EGFP; Lane 4: Hairy roots expressing (SP)_32_-EGFP. Normally Hyp-*O*-glycosylated (SP4)_18_-EGFP and non-glycosylated (SP4)_18_-EGFP products is indicated by while and grey arrow, respectively. Non-Hyp-*O*-glycosylated (SP)_32_-EGFP products (40-42 kDa) is indicated by dark arrow.


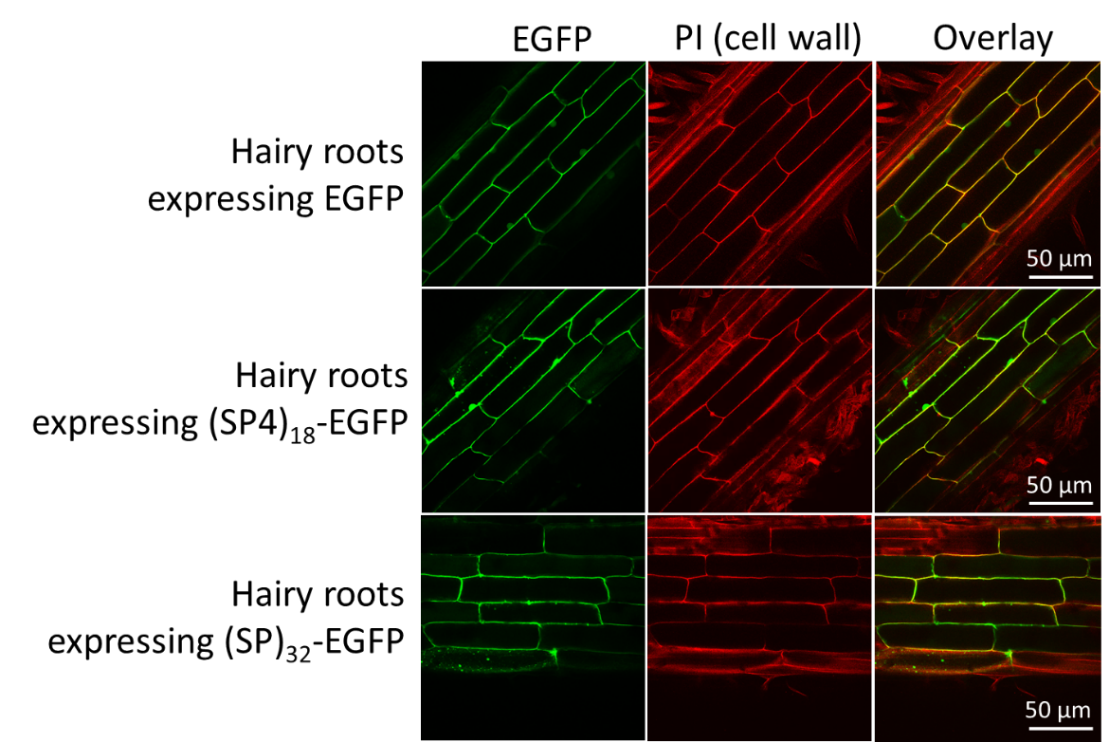


**Figure S4. Confocal laser scanning microscopy images of hairy roots expressing EGFP, (SP4)_18_-EGFP and (SP)_32_-EGFP.** The hairy roots were grown in petri dish for one week before the EGFP green fluorescence was inspected using a confocal microscope with a 40x/0.8W water-immersion objective. The cell wall of the root tissues was stained with propidium iodide (PI) red fluorescence for 1 min before the detection. The samples were excited and detected at the following wavelengths: 543 nm excitation with 595/50 nm filter for PI red fluorescence and 488 nm excitation with 525/50 nm filter for EGFP green fluorescence. Scale bar =50 µm.

**
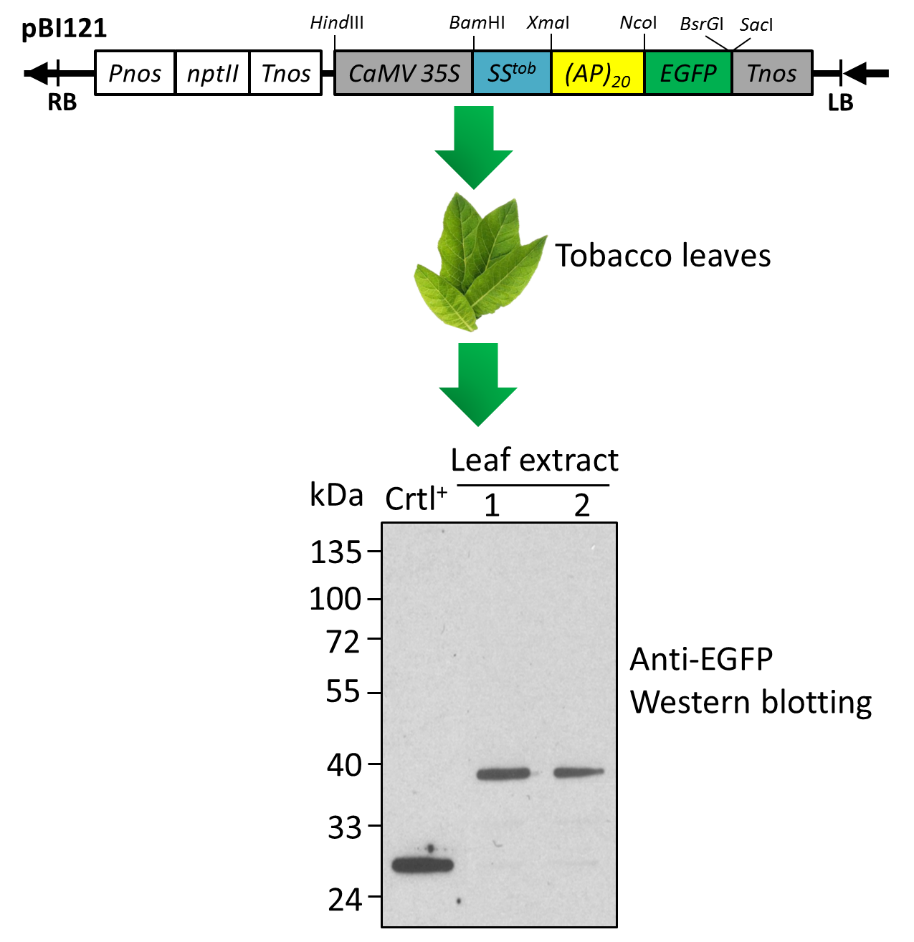
**

**Figure S5. Expression of (AP)_20_-EGFP in tobacco plants and anti-EGFP Western blotting detection of the transgene products accumulated in tobacco leaves.** Synthetic gene encoding 20 tandem repeats of “Ala-Pro” or (AP)_20_ was expressed as a fusion with EGFP in tobacco (*Nicotiana tabacum* L. var Xanthi). Soluble proteins extracted from the leaves of two transgenic plants (Lane 1 and 2) were used for anti-EGFP Western blotting assay. Ten µl of clarified leaf extracts was loaded into each well. Crtl^+^: EGFP standard (50 ng).


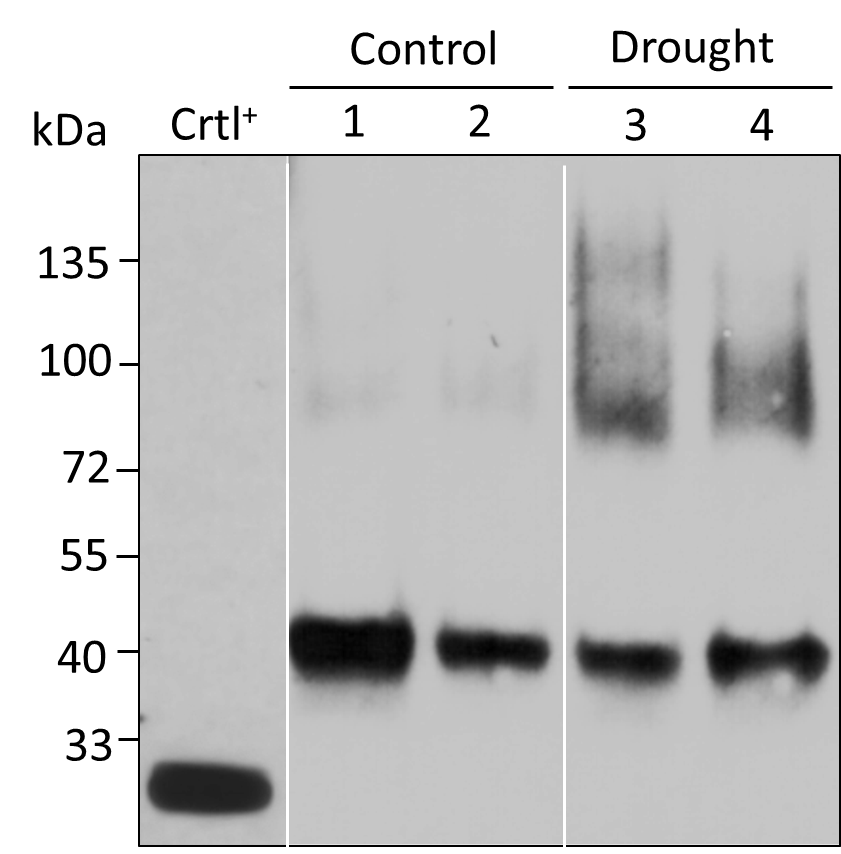


**Figure S6. Anti-EGFP Western blotting detection of the *(SP)_32_-EGFP* transgene products in the leaves of tobacco plants under drought stress**. Two transgenic tobacco lines (Lane 3 and 4) were grown in soils for 15 days with regular watering (every 3 days) before drought stress was applied (water was withheld). The *(SP)_32_-EGFP* transgene products accumulated in plant leaves were detected 9 days post drought treatment (day 24). Two control plants (Lane 1 and 2) were watered every three days for 24 days; The Hyp-*O*-glycosylation of the (SP)_32_-EGFP polypeptide was triggered in the plants subjected to drought stress, leading to the generation of the Hyp-*O*-glycosylated products (80 to 115 kDa). Ten µl of clarified leaf extracts was loaded into each well. Crtl^+^: EGFP standard (50 ng).

**Appendix S1**

**Generation of stably transformed tobacco plantlets**

*pBI121* expression vectors harboring the *EGFP*, *(SP)_32_-EGFP* or *(SP4)_18_-EGFP* gene constructs were each mobilized into *Agrobacterium tumefaciens* strain LBA4404 using the freeze/thaw method, and then transformed into tobacco (*Nicotiana tabacum* L*.* var Xanthi) plants using the *Agrobacterium-*mediated leaf-disk method (Horsch et al., 1988). Shoots grown from the leaf disks on the selection media consisting of Murashige and Skoog (MS) salts, 30 g/L sucrose, 1.0 mg/L 6-benzylaminopurine, 0.1 mg/L α-naphthaleneacetic, 200 mg/L kanamycin and 200 mg/L timentin) were excised and subcultured at 25 °C under continuous white light in Magenta^TM^ boxes. At least four high-expression transformants for each construct were selected from more than 30 transformants using anti-EGFP Western blotting assay (data not shown). The selected plantlets were grown in plant growth regulator-free MS medium for rooting.

**Appendix S2**

**Hairy root culture and determination of root tissue biomass and growth rate**

Transgenic hairy roots were maintained in Petri dishes containing SH medium (SH basal salts supplemented with 34 g/L of sucrose), and subcultured every 3 to 4 weeks. Liquid cultures of hairy roots were conducted in 250-mL Erlenmeyer flasks containing 50 ml SH medium, and agitated on a gyratory shaker rotated at 90 rpm at room temperature. Subcultures were carried out every 14 to 16 days by inoculating 15 root tips of ~1.5 cm in length in each flask (~0.2 to 0.25 g fresh weight). For kinetic studies of the root growth and protein production/secretion, cultured hairy roots and medium samples were collected every three days over a period of 21 days for determining the root biomass and the yields of secreted and intracellular EGFP. Parts of the collected root tissues and medium samples were also subject to anti-EGFP Western blotting assay. For measuring the root biomass, cultured hairy roots were harvested by vacuum filtration, washed three times with distilled water, and then freeze-dried in a lyophilizer for 48 hr to determine the dry weight (DW). Specific cell growth rate (*µ)* was estimated as described previously (Xu et al., 1998).

**Appendix S3**

**Expression of (SP)_32_-EGFP and (SP4)_18_-EGFP in *E. coli***

Gene fragments *(SP)_32_-EGFP* and *(SP4)_18_-EGFP* was each amplified by PCR using the In-Fusion^®^ cloning primer pair: 5’-AGGAGATATACCATGGCACAAACAACCCGGGCCTC-3’ and 5’-GACGGAGCTCGAATTCTTACTTGTACAGCTCGTCC-3’. The amplified gene fragment was subcloned into the *pET28b(+)* expression vector with the In-Fusion^®^ HD Cloning Kit (Takara Bio USA, Inc., CA) to generate the vectors *pET28b-(SP)_32_-EGFP* and *pET28b-(SP4)_18_-EGFP*, respectively, which were then delivered into the *E. coli* BL21 (DE3) strain. Gene expression was driven by the T7 promoter. The transformed *E. coli* was cultured in Luria-Bertani (LB) medium at 37 °C until OD_600_ reached ~0.6, and then induced for gene expression by adding 1 mM IPTG. After three hours the cultured cells were harvested by centrifugation and resuspended in the lysis buffer provided by the Ni-NTA Fast Start Kit (Qiagen, CA). The cell lysate was centrifuged at 12,000 ×g for 10 min and the supernatant was collected and stored at -20 °C for Western blotting assay.

**Appendix S4**

**SDS-PAGE and Western blotting assay**

Samples and EGFP standard (BioVision, Milpitas, CA) were separated on a 10% or 4-20% Tris-HCl gel using a Mini-Protean cell (Bio-Rad, Hercules, CA). After electrophoresis, proteins were electro-blotted onto a 0.2 μm nitrocellulose membrane (Bio-Rad, CA). Protein blots were blocked with 3% (w/v) BSA in Tris-buffered saline buffer (pH 7.5) containing 0.1% Tween^®^ 20 for 1.0 hr at room temperature. Immunoblot detection of EGFP was carried out using a rabbit anti-EGFP antibody (ThermoFisher Scientific, Waltham, MA) as primary antibody and a peroxidase-conjugated goat anti-rabbit IgG (Jackson ImmunoResearch Labs, PA) as secondary antibody. Protein blots were then detected using the SuperSignal® West Pico Chemiluminescent Substrate (ThermoFisher Scientific) in accordance with manufacturers’ procedures. EZ-Run™ Prestained Rec Protein Ladder (ThermoFisher Scientific) was used as a molecular weight marker for Western blots and Coomassie Brilliant Blue R-250-stained gels.

**References**

Bergman, T., Carlquist, M. and Jornvall, H. (1986) Amino acid analysis by high performance liquid chromatography of phenylthiocarbamyl derivatives. In: *Advanced Methods in Protein Microsequence Analysis* (Wittmann-Liebold, B. ed), pp 45–55. Berlin: Springer.

Horsch, R.B., Fry, J., Hoffmann, N., Neidermeyer, J., Rogers, S.G. and Fraley, R.T. (1988) Leaf disc transformation. In: *Plant Molecular Biology Manual* (Gelvin, S.B. and Schilperoort, R.A. eds), pp 1-9. Dordrecht: Kluwer Academic Publishers.

Oka, T., Saito, F., Shimma, Y., Yoko-o, T., Nomura, Y., Matsuoka, K. and Jigami, Y. (2010) Characterization of endoplasmic reticulum-localized UDP-D-galactose: hydroxyproline O-galactosyltransferase using synthetic peptide substrates in Arabidopsis. *Plant Physiology* **152**, 332-340.

Shpak, E., Barbar, E., Leykam, J.F. and Kieliszewski, M.J. (2001) Contiguous hydroxyproline residues direct hydroxyproline arabinosylation in Nicotiana tabacum. *The Journal of Biological Chemistry* **276**, 11272-11278.

Tan, L., Leykam, J.F. and Kieliszewski, M.J. (2003) Glycosylation motifs that direct arabinogalactan addition to arabinogalactan-proteins. *Plant Physiology* **132**, 1362-1369.

Tan, L., Varnai, P., Lamport, D.T., Yuan, C., Xu, J., Qiu, F. and Kieliszewski, M.J. (2010) Plant O-hydroxyproline arabinogalactans are composed of repeating trigalactosyl subunits with short bifurcated side chains. *The Journal of Biological Chemistry* **285**, 24575-24583.

Xu, J., Okada, S., Tan, L., Goodrum, K.J., Kopchick, J.J. and Kieliszewski, M.J. (2010) Human growth hormone expressed in tobacco cells as an arabinogalactan-protein fusion glycoprotein has a prolonged serum life. *Transgenic Research* **19**, 849-867.

Xu, J., Su, Z.G. and Feng, P.S. (1998) liquid culture of compact callus aggregate of Rhodiola sachalinensis for improved salidroside production. *Enzyme and Microbial Technology* **23**, 20-27.

Xu, J., Tan, L., Lamport, D.T.A., Showalter, A.M. and Kieliszewski, M.J. (2008) The O-Hyp glycosylation code in tobacco and Arabidopsis and a proposed role of Hyp-glycans in secretion. *Phytochemistry* **69**, 1631-1640.

Zhang, N., Gonzalez, M., Savary, B. and Xu, J. (2016) High-yield secretion of recombinant proteins expressed in tobacco cell culture with a designer glycopeptide tag: Process development. *Biotechnology Journal* **11**, 497-506.
